# FIS1 dependent peripheral fission exacerbates mitochondrial fragmentation and dysfunction in prion diseases

**DOI:** 10.3389/fnins.2026.1893232

**Published:** 2026-08-05

**Authors:** Fengting Gou, Zhiping Li, Jie Li, Dongming Yang, Mengyang Zhao, Dongdong Wang, Pei Wen, Zhixin Sun, Jingjing Wang, Qing Fan, Yuexin Dai, Deming Zhao, Lifeng Yang

**Affiliations:** State Key Laboratory of Veterinary Public Health and Safety, Key Laboratory of Animal Epidemiology of the Ministry of Agriculture and Rural Affairs, National Animal Transmissible Spongiform Encephalopathy Laboratory, College of Veterinary Medicine, China Agricultural University, Beijing, China

**Keywords:** DRP1, FIS1, mitochondria–lysosome contacts, peripheral fission, prion diseases

## Abstract

**Introduction:**

Mitochondrial dysfunction is an early and critical feature of neuronal injury in prion diseases, a group of fatal transmissible neurodegenerative disorders. Mitochondrial dynamics, a core component of mitochondrial quality control, maintains neuronal homeostasis through balanced fission and fusion. Although mitochondrial fission can be further divided into distinct spatial subtypes, the specific subtype involved in prion-associated neurotoxicity and its regulatory mechanisms remain unclear. This study aimed to identify the mitochondrial fission subtype involved in prion toxicity and elucidate its underlying regulatory mechanisms.

**Methods:**

Using live-cell time-lapse imaging, we analyzed mitochondrial fission dynamics in mouse neuroblastoma (N2a) cells treated with the neurotoxic prion peptide PrP^106−126^. Molecular mechanisms regulating mitochondrial peripheral fission were investigated through protein interaction analysis, mitochondrial-lysosome contact assessment, DRP1 activity analysis, FIS1 knockdown, and pharmacological inhibition using P110.

**Results:**

PrP^106−126^ selectively enhanced mitochondrial peripheral fission, identifying this subtype as a key pathological event underlying early mitochondrial damage. FIS1 acted as a central adaptor regulating this aberrant process by recruiting TBC1D15 to promote RAB7 GTP hydrolysis, thereby destabilizing mitochondria-lysosome contacts. In parallel, FIS1 recruited activated DRP1 to mitochondria. PrP^106−126^ treatment increased DRP1 Ser616 phosphorylation, decreased DRP1 Ser637 phosphorylation, and enhanced DRP1 GTPase activity, which were required for mitochondrial peripheral fission. FIS1 knockdown and P110-mediated disruption of the DRP1-FIS1 interaction effectively suppressed PrP^106−126^-induced peripheral mitochondrial fission, restored mitochondrial integrity, and reduced neuronal apoptosis.

**Discussion:**

These findings define mitochondrial peripheral fission as a key early pathogenic event in prion toxicity and identify FIS1 as a potential therapeutic target for intervention in prion diseases.

## Introduction

Prion diseases comprise chronic, progressive, and lethal infectious neurodegenerative conditions that impact both humans and various mammalian species (Zerr et al., 2024; Galassi et al., 2016). These diseases are characterized by several histopathological features, such as spongiform encephalopathy, gliosis, neuronal loss, and scrapie prion protein isoform (PrP^Sc^) deposition (Sigurdson et al., 2019). Prion pathogenesis arises when the normal cellular prion protein (PrP^C^) misfolds into the pathogenic PrPSc form, which contains more β-sheet structures and a protease-resistant core (Bolton et al., 1982; Prusiner, 1982). The prion protein (PrP) fragment PrP^106−126^ is a neurotoxic peptide homologous to residues 106–126 of human PrP. PrP^106−126^ remains highly conserved among different species and serves as an essential domain for triggering the conformational conversion of PrP^C^ into PrP^Sc^ (Corsaro et al., 2003; Thellung et al., 2018; Florio et al., 2003; Forloni et al., 1993; Gu et al., 2002; Jeong et al., 2017, 2011; Thellung et al., 2000). PrP^106−126^ retains most of the pathogenic properties of PrP^Sc^, including neurotoxicity, apoptosis induction, and autophagy, making it widely used in prion disease neurotoxicity models.

Neurons are highly sensitive to mitochondrial function due to their high energy consumption (Cheng et al., 2022). Since mitochondria play a pivotal role in cellular metabolism and intracellular signaling, their dysfunction may contribute to the development of multiple neurodegenerative disorders. Proper mitochondrial function is maintained by a complex system of quality control pathways, including mitochondrial autophagy, biogenesis, and dynamics (Picca et al., 2018; Roca-Portoles and Tait, 2021; Xian and Liou, 2021; Dong et al., 2023). Mitochondrial dynamics, which include fusion, fission, and transport, significantly impact various physiological processes, such as ATP production regulation in response to energy demands and coordination of complex cell signaling events (Chan, 2020; Chen et al., 2023; Zacharioudakis and Gavathiotis, 2023). Mitochondrial fission is a tightly controlled event associated with cellular proliferation, metabolic activity, and apoptosis, primarily mediated by dynamin-related protein 1 (DRP1). DRP1 is composed of an N-terminal GTPase domain, a dynamin-like middle domain, a variable region, and a C-terminal GTPase effector domain. It is widely expressed in mammals and highly conserved across evolution. During mitochondrial fission, DRP1 is recruited to the outer mitochondrial membrane (OMM) by various receptors, where it hydrolyzes GTP to constrict and sever mitochondria (Mozdy et al., 2000; Wang et al., 2020; Kraus et al., 2021). Mitochondrial fission is subdivided into midzone and peripheral fission, both mediated by DRP1. If the fission site is located within 25% of either end of the mitochondrion, it is classified as peripheral fission. If the fission site is at the 50% midpoint, it is classified as midzone fission. The pre-constriction process, coordinated by the endoplasmic reticulum, actin filaments, and the adaptor protein MFF, selectively controls mitochondrial midzone fission involved in mitochondrial biogenesis. However, the OMM protein Mitochondrial fission protein 1 (FIS1) controls mitochondrial peripheral fission, which separates damaged material for mitochondrial autophagy and occurs following mitochondria-lysosome contact (Wong et al., 2018; Kleele et al., 2021).

Multiple neurodegenerative disorders, such as Parkinson's disease (PD) and Alzheimer's disease (AD), are related to neuronal mitochondrial impairment associated with DRP1-mediated fission (Morton et al., 2021; Xi et al., 2025). Prior research has shown that DRP1-dependent mitochondrial fission aggravates neuronal mitochondrial fragmentation in both *in vivo* and *in vitro* prion disease models (Li et al., 2018). Given the complex physiological effects of DRP1-mediated mitochondrial fission, this study aimed to identify the predominant type of fission exacerbated in prion diseases and elucidate its underlying mechanism to achieve more precise regulation of mitochondrial dynamics. Our findings suggest that FIS1 mediates excessive PrP^106−126^-induced mitochondrial peripheral fission in mouse neuroblastoma (N2a) cells, exacerbating mitochondrial fragmentation and dysfunction. FIS1 promotes the untethering of mitochondria-lysosome contacts by recruiting Tre-2/Bub2/Cdc16 (TBC) domain family member 15 (TBC1D15) to hydrolyze RAB7 and recruits active DRP1 to mediate mitochondrial peripheral fission in prion disease models. Inhibition of FIS1-mediated mitochondrial peripheral fission alleviates PrP^106−126^-induced mitochondrial morphological damage and dysfunction and reduces neuronal apoptosis. Our study provides a potentially more precise therapeutic target for prion and other neurodegenerative diseases.

## Results

### PrP^106−126^ exacerbates mitochondrial peripheral fission in N2a cells

**Figure 1:**
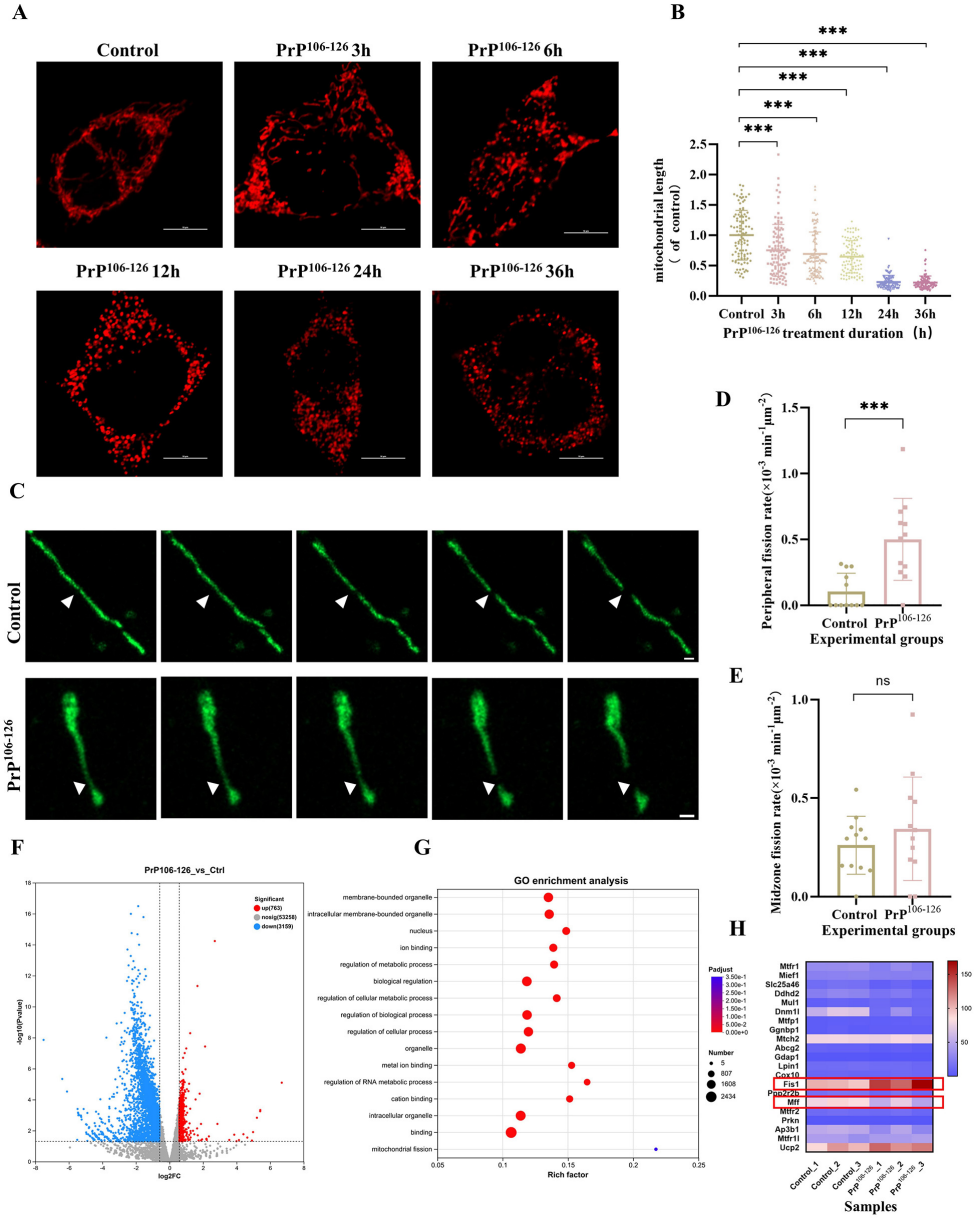
PrP^106−126^ exacerbates mitochondrial peripheral fission in N2a cells. **(A)** Confocal microscopy was used to measure mitochondrial morphology in N2a cells treated with PrP^106−126^. Scale bar: 10 μm. **(B)** Comparison of mitochondrial length in N2a cells under different durations of PrP^106−126^ treatments. **(C)** Time-lapse imaging of mitochondrial peripheral and midzone fission in N2a cells. Mitochondria were stained with Mito-Tracker Green. Scale bar: 1 μm. **(D)** Peripheral fission rates in N2a cells. **(E)** Midzone fission rates in N2a cells. **(F)** Volcano map of DEGs between PrP^106−126^-treated and the control N2a cells. **(G)** Gene ontology enrichment analysis between PrP^106−126^-treated and the control N2a cells. **(H)** Heatmap of gene mRNA expression in the mitochondrial fission pathway. The data are presented as the mean ± SD. ns, not significant; ***P < 0.001. *n* = at least three biologically independent treatments of cells for each. For live-cell imaging analysis of mitochondrial fission, five cells were analyzed per biological replicate.

Previous laboratory results suggest that mitochondria in prion disease models exhibit fragmentation, which is associated with increased mitochondrial fission (Li et al., 2018). Since mitochondrial fission is classified into midzone and peripheral fission, we aimed to explore how these two types of fission are altered in prion disease models (Kleele et al., 2021). In a preliminary experiment, we treated N2a cells with different concentrations of PrP^106−126^ and found that cell viability decreased with 100 μM PrP^106−126^ treatment, but remained unchanged with 100 μM scrambled peptide (Supplementary Figure S1). Therefore, we selected 100 μM PrP^106−126^ for the subsequent experiments. To assess mitochondrial morphology after prolonged PrP^106−126^ treatment (3, 6, 12, 24, and 36 h), we used confocal microscopy to measure mitochondrial length. The control group showed an intact mitochondrial network, whereas mitochondrial length decreased with prolonged PrP^106−126^ incubation (Figures 1A, B). To identify the type of fission associated with mitochondrial fragmentation, we employed live-cell time-lapse imaging to observe all mitochondria in N2a cells. We found that the mitochondrial peripheral fission rate increased while the midzone fission remained unchanged in PrP^106−126^-treated N2a cells, compared to the control group (Figures 1C–E). To investigate the mechanisms of mitochondrial fission, we used transcriptome sequencing and identified a total of 3922 differentially expressed genes (DEGs) between PrP^106−126^-treated and control N2a cells, with 3159 downregulated and 763 upregulated (Figure 1F). Gene ontology enrichment analysis of the DEGs revealed that the mitochondrial fission pathway was enriched (Figure 1G). Further examination of DEGs in the mitochondrial fission pathway revealed that FIS1, which mediates peripheral fission, exhibited elevated transcriptional levels, while MFF, which regulates midzone fission, remained unchanged (Figure 1H). These results collectively suggest that PrP^106−126^ treatment exacerbates mitochondrial peripheral fission in N2a cells.

### FIS1 mediates mitochondrial peripheral fission in PrP^106−126^-treated N2a cells

**Figure 2:**
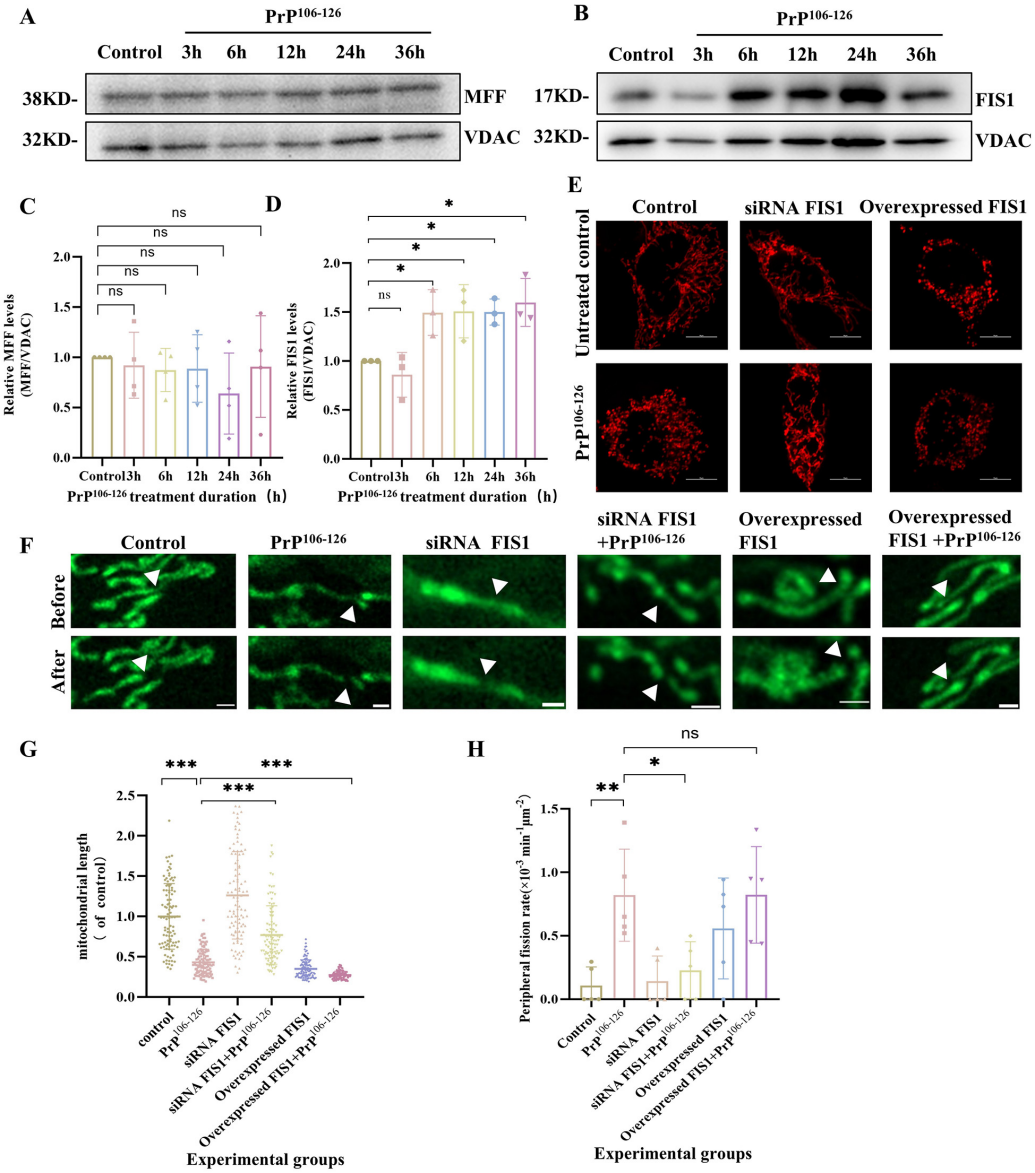
FIS1 mediates mitochondrial peripheral fission in PrP^106−126^-treated N2a cells. **(A)** Western blotting of mitochondrial MFF protein extracted from N2a cells. VDAC was used as the loading control. **(B)** Western blotting of mitochondrial FIS1 protein extracted from N2a cells. VDAC was used as the loading control. **(C)** Comparison of MFF protein levels, relative to control. **(D)** Comparison of FIS1 protein levels, relative to control. **(E)** Confocal microscopy was used to measure mitochondrial morphology in knocked down (siRNA) FIS1 or overexpressed FIS1 cells with and without PrP^106−126^ treatment. Scale bar: 10 μm. **(F)** Time-lapse imaging of mitochondrial peripheral fission in knocked down (siRNA) FIS1 or overexpressed FIS1 cells with and without PrP^106−126^ treatment. Mitochondria were stained with Mito-Tracker Green. Scale bar: 1 μm. **(G)** Comparison of mitochondrial length in the different treatment groups. **(H)** Peripheral fission rates in N2a cells. The data are presented as the mean ± SD. ns, not significant; *P < 0.05; **P < 0.01; ***P < 0.001. *n* = at least three biologically independent treatments of cells for each. For live-cell imaging analysis of mitochondrial fission, five cells were analyzed per biological replicate.

Literature reports indicate that peripheral mitochondrial fission is mediated by FIS1, while midzone fission is driven by MFF (Kleele et al., 2021). We aim to further investigate the mechanisms underlying altered mitochondrial fission in prion disease models. Western blotting showed that mitochondrial MFF protein levels remained unchanged (Figures 2A, C), whereas FIS1 protein levels increased (Figures 2B, D) in N2a cells with prolonged incubation of PrP^106−126^ (6, 12, 24, and 36 h). To examine whether these molecular changes are conserved in another neuronal cell type, we performed the same analyses in human SH-SY5Y neuroblastoma cells. Similar to N2a cells, mitochondrial FIS1 expression increased over time following PrP^106−126^ treatment, whereas MFF expression remained unchanged (Supplementary Figures S2A–D), demonstrating that the differential regulation of FIS1 and MFF is reproducible in an independent neuronal cell line. To investigate whether FIS1 regulates peripheral fission in an *in vitro* prion disease model, we transfected N2a cells with siRNA targeting FIS1 (Supplementary Figures S2E, F) or FIS1 overexpression plasmid (Supplementary Figures S2G, H). Confocal microscopy results showed that knocking down FIS1 expression alleviated PrP^106−126^-induced mitochondrial fragmentation (Figures 2E, G), suggesting that FIS1 regulates mitochondrial morphology in prion disease. We then used live-cell time-lapse imaging to investigate whether modulating FIS1 alters peripheral fission and found that inhibition of FIS1 reduced the excessive PrP^106−126^-induced mitochondrial peripheral fission in N2a cells (Figures 2F, H). These results suggest that FIS1 on the mitochondrial membrane mediates the excessive peripheral fission in PrP^106−126^-treated N2a cells.

### FIS1 recruits TBC1D15 to mitochondria in PrP^106−126^-treated N2a cells

**Figure 3:**
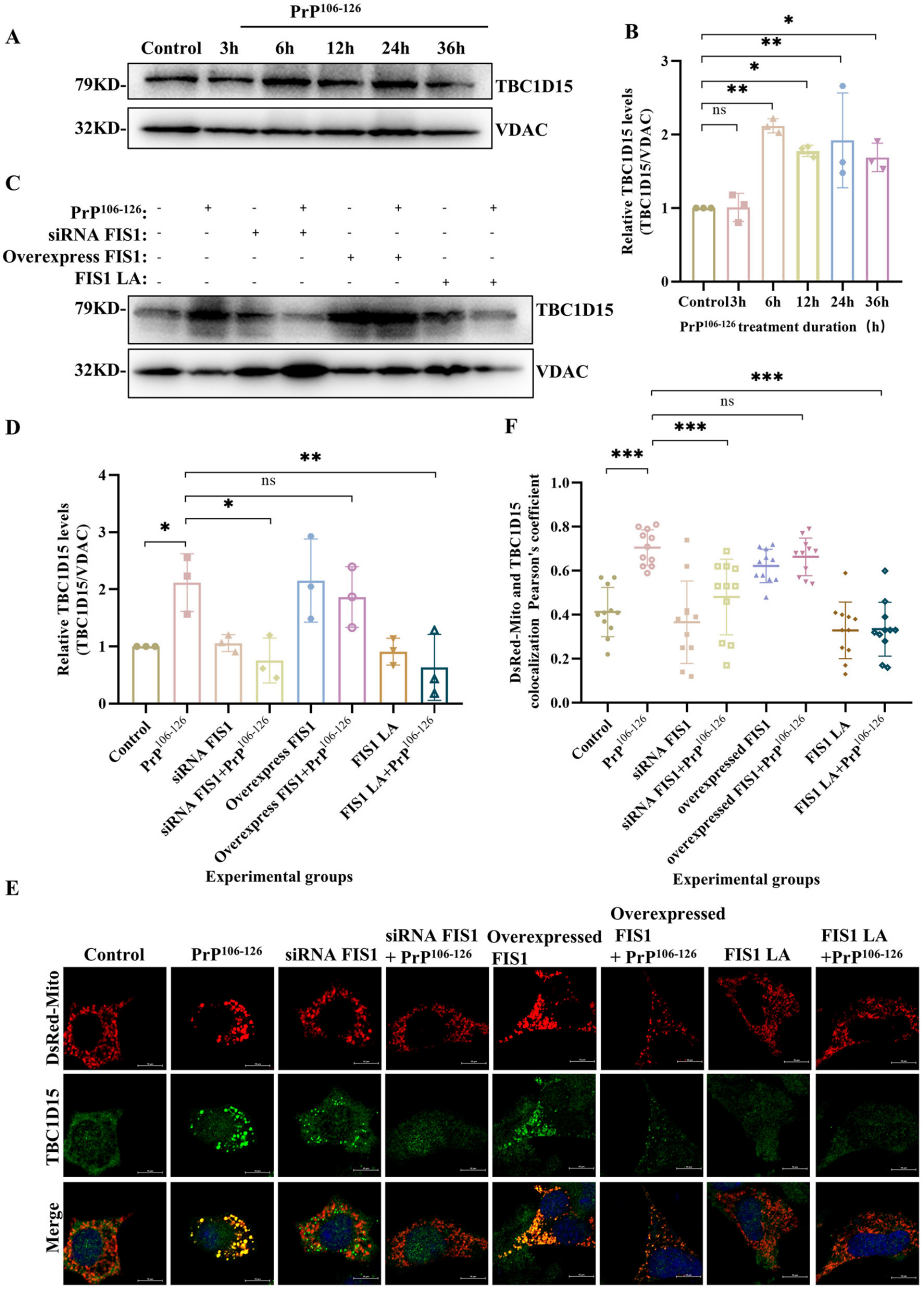
FIS1 recruits TBC1D15 to mitochondria in PrP^106−126^-treated N2a cells. **(A)** Western blotting of mitochondrial TBC1D15 protein extracted from N2a cells. VDAC was used as the loading control. **(B)** Comparison of TBC1D15 protein levels, relative to control. **(C)** Western blotting of mitochondrial TBC1D15 protein extracted from N2a cells. VDAC was used as the loading control. **(D)** Comparison of TBC1D15 protein levels, relative to control. **(E)** Immunofluorescence co-localization detection of the mitochondrial marker DsRed-Mito and TBC1D15 in knocked down (siRNA) FIS1, overexpressed FIS1, and FIS1 LA cells with or without PrP^106−126^ treatment. Scale bar: 10 μm. **(F)** Comparison of the localization of TBC1D15 and DsRed-Mito in cells. The data are presented as the mean ± SD. ns, not significant; *P < 0.05; **P < 0.01; ***P < 0.001. *n* = at least three biologically independent treatments of cells for each.

Next, we investigated the specific mechanisms by which FIS1 mediates mitochondrial peripheral fission in prion disease models. The C-terminus of FIS1 contains a transmembrane structural domain that anchors it to the mitochondria, while the N-terminus contains a triangular tetrapeptide repeat-like domain that regulates mitochondrial fission by recruiting TBC1D15 to the mitochondria (Onoue et al., 2013; Ihenacho et al., 2023). Therefore, we aimed to investigate whether FIS1 recruits TBC1D15 to mediate mitochondrial peripheral fission in a prion disease model. Compared to the control group, TBC1D15 protein level increased after 6–36 h of incubation with PrP^106−126^ (Figures 3A, B). To further investigate the association between elevated TBC1D15 levels and FIS1, we transfected N2a cells with FIS1 siRNA, FIS1 overexpression plasmid, or a FIS1 LA mutant plasmid, which cannot recruit TBC1D15 (Jofuku et al., 2005). The efficiency of FIS1 knockdown and overexpression was confirmed (Supplementary Figures S2E–H), and the expression of the FIS1 LA mutant was validated (Supplementary Figures S3A, B) in N2a cells. Western blot results showed that knocking down FIS1 or expressing the FIS1 LA mutant alleviated elevated TBC1D15 protein levels on mitochondria following PrP^106−126^ treatment (Figures 3C, D). Similarly, immunofluorescence results revealed that mitochondria and TBC1D15 co-localization significantly increased upon PrP^106−126^ treatment. Furthermore, this elevated co-localization was reduced with FIS1 knockdown or FIS1 LA expression (Figures 3E, F), indicating that FIS1 recruits TBC1D15 to the mitochondria after PrP^106−126^ treatment in N2a cells. These results demonstrate that FIS1 plays a key role in recruiting TBC1D15 to mitochondria in the prion disease model.

### TBC1D15 hydrolyzes RAB7 GTP promoting mitochondrial peripheral fission in PrP^106−126^-treated N2a cells

**Figure 4:**
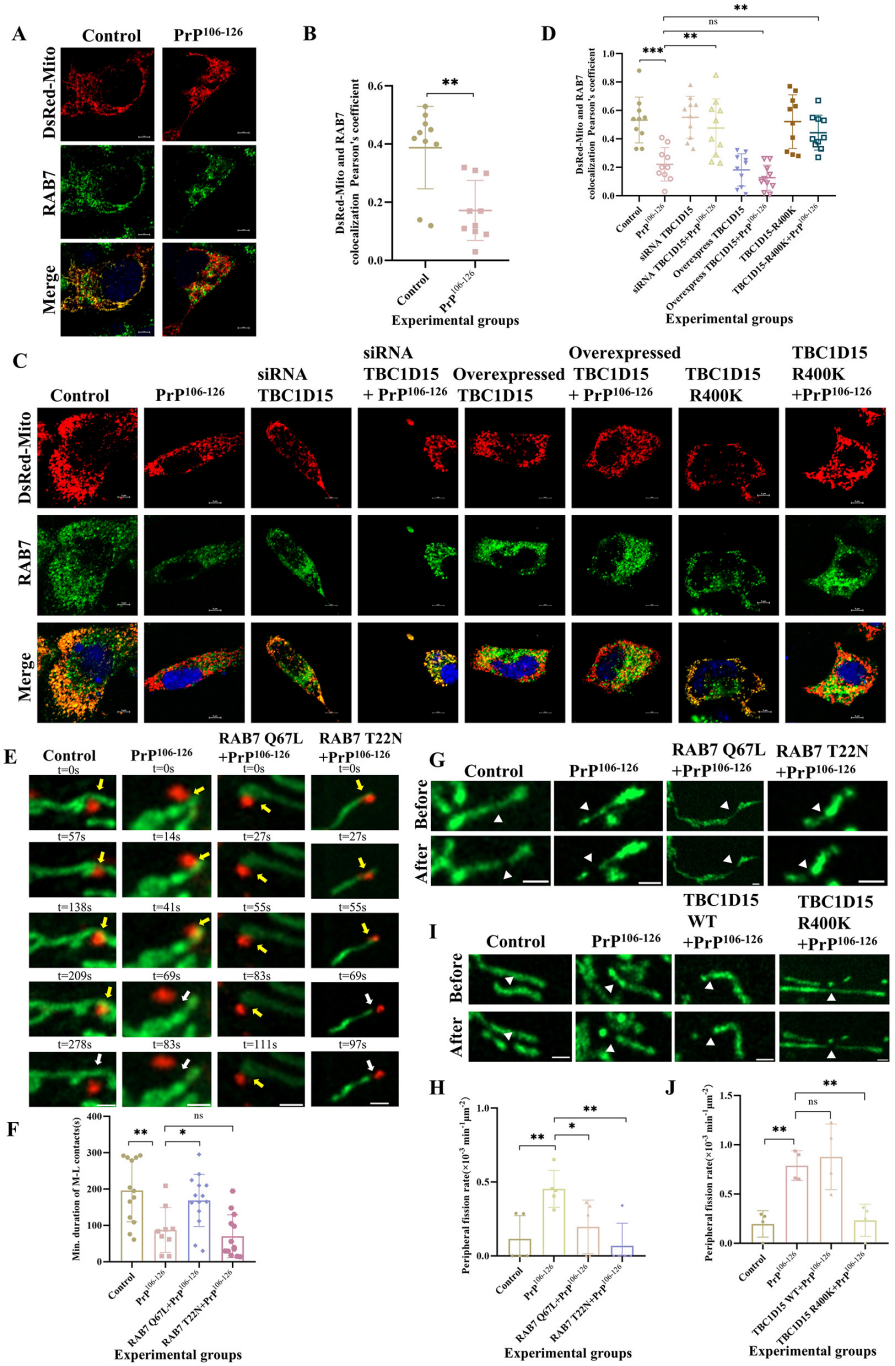
TBC1D15 hydrolyzes RAB7 GTP promoting mitochondrial peripheral fission in PrP^106−126^-treated N2a cells. **(A)** Immunofluorescence co-localization detection of the mitochondrial marker DsRed-Mito and RAB7 in N2a cells with or without PrP^106−126^ treatment. Scale bar: 5 μm. **(B)** Comparison of the localization of RAB7 and DsRed-Mito in N2a cells. **(C)** Immunofluorescence co-localization detection of the mitochondrial markers DsRed-Mito and RAB7 in knocked down (siRNA) TBC1D15, overexpressed TBC1D15, and TBC1D15 R400K cells with or without PrP^106−126^ treatment. Scale bar: 5 μm. **(D)** Comparison of the localization of RAB7 and DsRed-Mito in N2a cells. **(E)** Representative time-lapse images of lysosome approaching mitochondria to form a stable contact (yellow arrows) before leaving mitochondria (white arrows) in overexpressed RAB7 Q67L and RAB7 T22N cells with or without PrP^106−126^ treatment. Mitochondria were labeled with Mito-Tracker Green, and lysosomes were labeled with Lyso-Tracker Red. Scale bar: 1 μm. **(F)** Quantification of minimum duration of mitochondria–lysosome contacts. **(G)** Time-lapse imaging of mitochondrial peripheral fission in overexpressed RAB7 Q67L and RAB7 T22N cells with or without PrP^106−126^ treatment. Mitochondria were stained with Mito-Tracker Green. Scale bar: 1 μm. **(H)** Peripheral fission rates in N2a cells. **(I)** Time-lapse imaging of mitochondrial peripheral fission in overexpressed TBC1D15 wild-type (WT) and TBC1D15 R400K cells with or without PrP^106−126^ treatment. Mitochondria were stained with Mito-Tracker Green. Scale bar: 1 μm. **(J)** Peripheral fission rates in N2a cells. The data are presented as the mean ± SD. ns, not significant; *P < 0.05; **P < 0.01; ***P < 0.001. *n* = at least three biologically independent treatments of cells for each. For live-cell imaging analysis of mitochondrial fission, five cells were analyzed per biological replicate.

Mitochondria-lysosome contacts regulate lysosomal morphology and serve as markers for sites of mitochondrial fission (Wong et al., 2018). TBC1D15 is a widely expressed cytoplasmic protein that, upon recruitment to the mitochondria, acts as a GTPase-activating protein for RAB7 (Zhang et al., 2005; Peralta et al., 2010). RAB7 GTP promotes the formation of mitochondria–lysosome contacts, whereas TBC1D15 facilitates the untethering of mitochondria–lysosome contacts by hydrolyzing RAB7 GTP to GDP. Therefore, we sought to elucidate whether TBC1D15 regulates mitochondria–lysosome contacts by enhancing hydrolysis of RAB7 GTP to mediate mitochondrial peripheral fission in a prion disease model. Since RAB7 GDP is released into the cytoplasm, we used immunofluorescence to observe the co-localization of RAB7 with mitochondria, which reflects RAB7 GTP levels. After 12 h of PrP^106−126^, the co-localization of mitochondria and RAB7 decreased, indicating that PrP^106−126^ induced a reduction in RAB7 GTP levels (Figures 4A, B). Next, we manipulated TBC1D15 using siRNA silencing (Supplementary Figures S4A, B), TBC1D15 overexpression (Supplementary Figures S4C, D), or the TBC1D15 R400K mutant (Supplementary Figures S4C, E), which cannot hydrolyze RAB7 GTP (Wong et al., 2018). The immunofluorescence results showed that knocking down TBC1D15 or expressing TBC1D15 R400K increased the co-localization of mitochondria and RAB7, suggesting that knocking down TBC1D15 or expressing TBC1D15 R400K could alleviated the PrP^106−126^-induced hydrolysis of RAB7 GTP (Figures 4C, D), which further demonstrates that TBC1D15 regulates RAB7 hydrolysis in the prion disease model. These experimental results indicate that TBC1D15 amplifies RAB7 GTP hydrolysis in PrP^106−126^-induced N2a cells.

To investigated mitochondria–lysosome contacts in a prion disease model, we manipulated RAB7 via RAB7 Q67L (active GTP-bound mutant), and RAB7 T22N mutants (inactive GDP-bound mutant) expression in N2a cells. Interestingly, live-cell time-lapse imaging showed that the duration of mitochondria-lysosome contacts decreased after PrP^106−126^ treatment, while RAB7 Q67L overexpression alleviated this reduction in contact duration (Figures 4E, F), suggesting that RAB7 GTP hydrolysis promotes the untethering of mitochondria-lysosome contacts in prion disease. We then examined the peripheral fission rate using live cell time-lapse imaging. However, overexpression of RAB7 Q67L and RAB7 T22N both reduced the peripheral fission rate (Figures 4G, H). This may be related to interference from RAB7 T22N with the formation of mitochondria-lysosome contacts. Therefore, we overexpressed wild-type (WT) or TBC1D15 R400K to regulate RAB7 hydrolysis on mitochondria. We found that TBC1D15 R400K alleviated the increased peripheral fission caused by PrP^106−126^, whereas TBC1D15 WT did not (Figures 4I, J), suggesting that the untethering of mitochondria–lysosome contacts regulate mitochondrial peripheral fission in prion disease. In conclusion, these results indicate that in PrP^106−126^-treated N2a cells, increased TBC1D15 amplifies RAB7 GTP hydrolysis on mitochondria, promoting the untethering of mitochondria-lysosome contacts and further facilitating mitochondrial peripheral fission.

### FIS1 on mitochondria recruits DRP1 in PrP^106−126^-treated N2a cells

**Figure 5:**
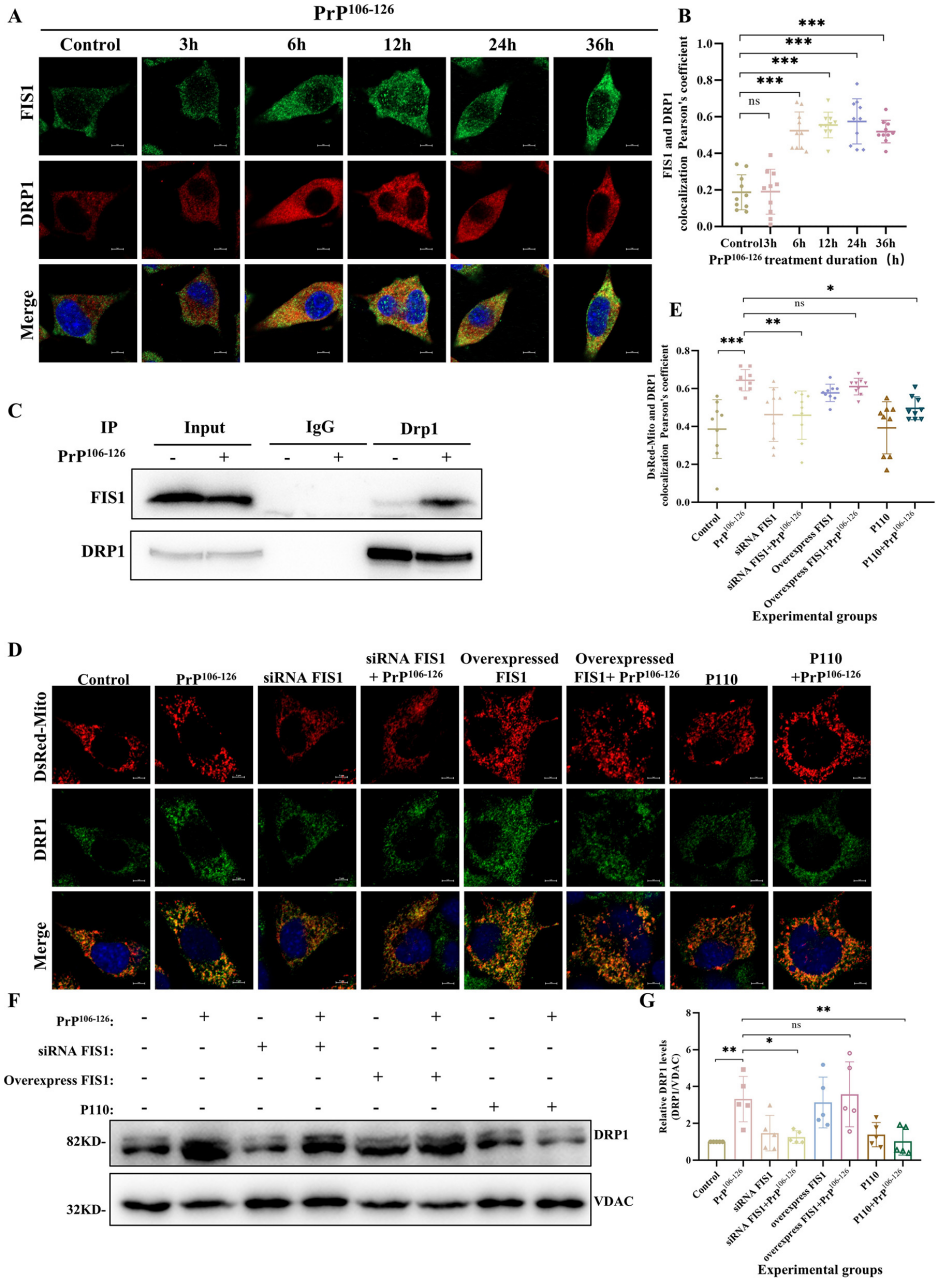
FIS1 on mitochondria recruits DRP1 in PrP^106−126^-treated N2a cells. **(A)** Immunofluorescence co-localization detection of FIS1 and DRP1 in N2a cells treated with different durations of PrP^106−126^. Scale bar: 5 μm. **(B)** Comparison of the localization of FIS1 and DRP1 in N2a cells. **(C)** N2a cells treated with PrP^106−126^ and their control were applied to co-IP assay with anti-Drp1 antibody. **(D)** Immunofluorescence co-localization detection of the mitochondrial marker DsRed-Mito and DRP1 in knocked down (siRNA) FIS1 and overexpressed FIS1 cells with or without PrP^106−126^ and P110 treatments. Scale bar: 5 μm. **(E)** Comparison of the localization of DRP1 and DsRed-Mito-labeled mitochondria in N2a cells. **(F)** Western blotting of mitochondrial DRP1 protein extracted from N2a cells. VDAC was used as the loading control. **(G)** Comparison of DRP1 protein levels, relative to control. The data are presented as the mean ± SD. ns, not significant; *P < 0.05; **P < 0.01; ***P < 0.001. *n* = at least three biologically independent treatments of cells for each.

**Figure 6:**
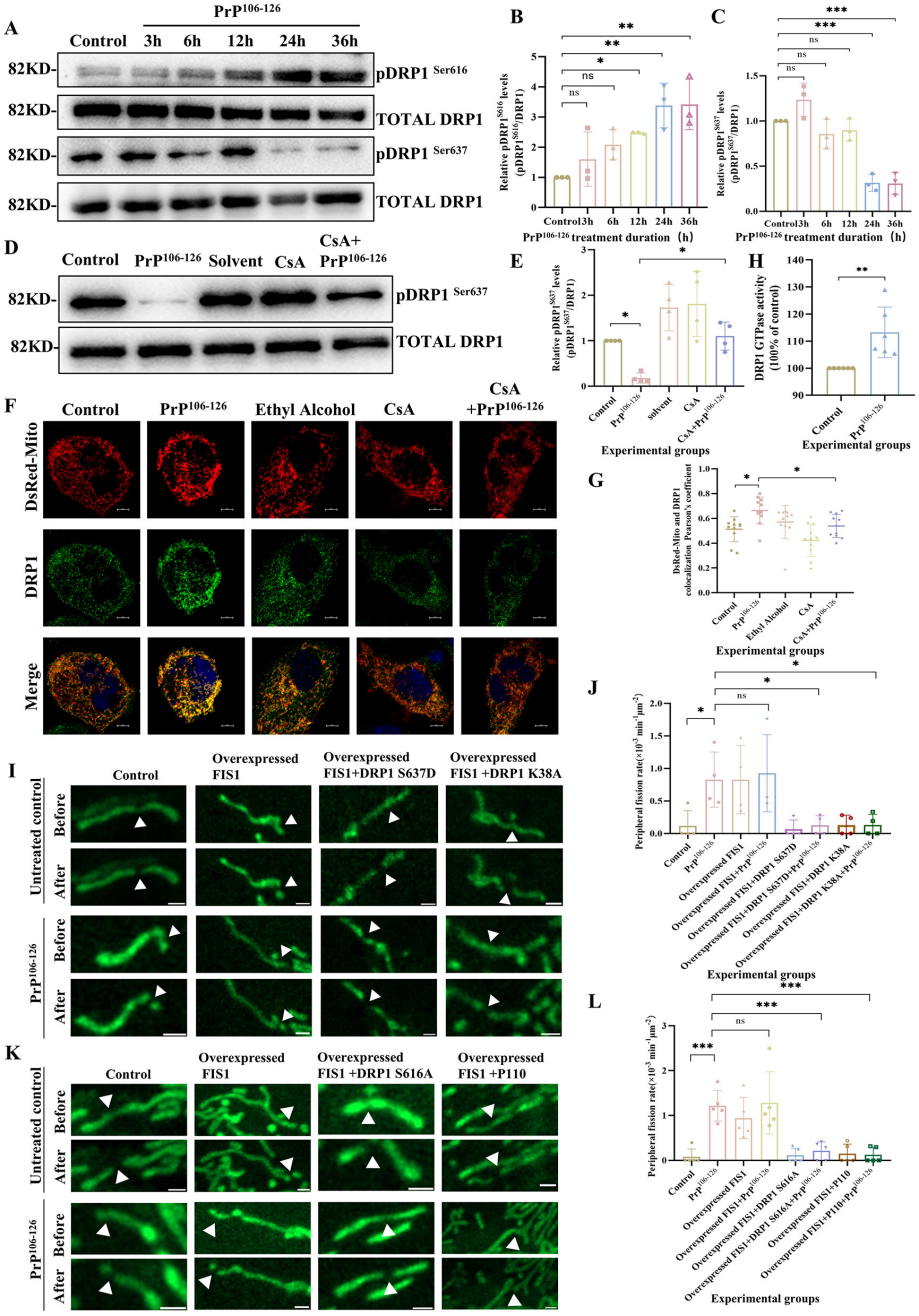
Activated DRP1 performs mitochondrial peripheral fission in the prion disease model. **(A)** Western blotting detection of phosphorylated DRP1 at sites S616 (pDRP1^S616^) and S637 (pDRP1^S637^) in N2a cells treated with or without PrP^106−126^. The total DRP1 protein was used as the loading control. **(B)** Comparison of pDRP1^S616^ protein levels, relative to control. **(C)** Comparison of pDRP1^S637^ protein levels, relative to control. **(D)** Western blotting detection of pDRP1^S637^ in N2a cells with or without PrP^106−126^ treatment. The total DRP1 protein was used as the loading control. **(E)** Comparison of pDRP1^S637^ protein levels, relative to control. **(F)** Immunofluorescence co-localization detection of the mitochondrial marker DsRed-Mito and DRP1 in N2a cells with or without PrP^106−126^ treatment. Scale bar: 5 μm. **(G)** Comparison of the localization of DRP1 and DsRed-Mito-labeled mitochondria in N2a cells. **(H)** DRP1 GTPase activity assay. **(I)** Time-lapse imaging of mitochondrial peripheral fission in overexpressed FIS1, DRP1 S637D, and DRP1 K38A cells with or without PrP^106−126^ treatment. Mitochondria were stained with Mito-Tracker Green. Scale bar: 1 μm. **(J)** Peripheral fission rates in N2a cells. **(K)** Time-lapse imaging of mitochondrial peripheral fission in overexpressed FIS1 and DRP1 S616A cells with or without PrP^106−126^ and P110 treatment. Mitochondria were stained with Mito-Tracker Green. Scale bar: 1 μm. **(L)** Peripheral fission rates in N2a cells. The data are presented as the mean ± SD. ns, not significant; *P < 0.05; **P < 0.01; ***P < 0.001. *n* = at least three biologically independent treatments of cells for each. For live-cell imaging analysis of mitochondrial fission, five cells were analyzed per biological replicate.

As reported in the literature, mitochondrial fission occurs following the untethering of mitochondria-lysosome contacts (Wong et al., 2018). Therefore, we aimed to determine whether DRP1 mediates peripheral fission after untethering of mitochondria-lysosome contacts in an *in vitro* prion disease model. We aimed to explore whether changes in DRP1 localization on mitochondria are related to FIS1. Immunofluorescence results showed that the co-localization of FIS1 and DRP1 increased in N2a cells after 6–36 h of PrP^106−126^ incubation (Figures 5A, B). Co-immunoprecipitation (co-IP) experiments further demonstrated that endogenous DRP1 strongly interacts with FIS1 in N2a cells after PrP^106−126^ treatment (Figure 5C). To confirm whether elevated DRP1 levels on mitochondria are regulated by FIS1, we transfected N2a cells with siRNA targeting FIS1 or a FIS1 overexpression plasmid, or treated them with 1 μM P110. P110 is a selective peptide inhibitor that blocks the interaction between DRP1 and FIS1 (Qi et al., 2012). Our cell viability assay showed that all concentrations of P110 (0.5, 1, 5, 10 μM) were non-toxic to N2a cells (Supplementary Figure S5A). Immunofluorescence results showed that co-localization of mitochondria and DRP1 in N2a cells increased significantly after PrP^106−126^ treatment, whereas the ratio of mitochondria to DRP1 co-localization decreased with FIS1 knockdown or P110 treatment (Figures 5D, E), suggesting that FIS1 regulates DRP1 translocation to mitochondria in prion disease. Similarly, Western blot results showed that inhibiting FIS1 expression or supplementing with P110 alleviated the PrP^106−126^-induced increase in DRP1 protein levels on mitochondria (Figures 5F, G). To investigate whether FIS1 regulates peripheral fission by recruiting DRP1, we treated N2a cells with P110. Live-cell time-lapse imaging showed that P110 relieved excessive mitochondrial peripheral fission induced by PrP^106−126^ in FIS1-overexpressing N2a cells (Figures 6K, L). Taken together, these findings suggest that FIS1 recruits DRP1 to mitochondria to mediate peripheral fission in prion disease models.

### Activated DRP1 performs mitochondrial peripheral fission in the prion disease model

The activated form of DRP1 plays a significant role in regulating mitochondrial fission. DRP1 activity is regulated by various post-translational modifications (PTMs), with phosphorylation at Ser616 and Ser637 being the most widely studied (Quiles and Gustafsson, 2022). Phosphorylation at Ser616 activates DRP1 to promote mitochondrial fission, while phosphorylation at Ser637 inhibits DRP1 activity, reducing mitochondrial fission (Chen et al., 2023). Therefore, we aimed to determine whether phosphorylation of DRP1 at Ser616 and Ser637 regulates mitochondrial peripheral fission in the prion disease model. We found that phosphorylation at DRP1 Ser616 increased after 6–36 h of PrP^106−126^ treatment (Figures 6A, B), while phosphorylation at Ser637 significantly decreased after 24–36 h (Figures 6A, C). To further evaluate whether DRP1 activation is conserved in another neuronal cell line, we examined DRP1 phosphorylation in SH-SY5Y cells following PrP^106−126^ treatment. Similar to N2a cells, PrP^106−126^ increased phosphorylation at Ser616 while decreasing phosphorylation at Ser637 in SH-SY5Y cells (Supplementary Figures S5B–E), indicating that DRP1 activation induced by PrP^106−126^ is reproducible in multiple neuronal cell models. Since FIS1 primarily functions as a mitochondrial outer membrane receptor for DRP1, we next investigated whether the activation state of DRP1 is required for mitochondrial peripheral fission. Next, we sought to determine how DRP1 phosphorylation affects mitochondrial peripheral fission. Phosphorylation of DRP1 at Ser637 is regulated by calcineurin. When calcineurin dephosphorylates DRP1 at Ser637, DRP1 translocates from the cytoplasm to the mitochondria, assembling into rings to facilitate mitochondrial fission (Cereghetti et al., 2008; Giacomello et al., 2020). To modulate DRP1 Ser637 phosphorylation, we treated cells with Cyclosporin A (CsA), a calcineurin inhibitor that blocks the dephosphorylation of DRP1 at Ser637 (Cereghetti et al., 2008). Cell viability assays showed that CsA concentrations of 1 and 2 μM were non-toxic to N2a cells (Supplementary Figure S6A). Western blot results showed that 2 μM CsA increased DRP1 Ser637 phosphorylation, indicating that CsA inhibited the PrP^106−126^-induced dephosphorylation of DRP1 at Ser637 (Figures 6D, E). Immunofluorescence results also showed that CsA treatment significantly reduced the co-localization of DRP1 and mitochondria after PrP^106−126^ treatment in N2a cells, suggesting that DRP1 Ser637 dephosphorylation is required for DRP1 translocation to the mitochondria (Figures 6F, G). To investigate whether phosphorylation at Ser616 or dephosphorylation at Ser637 affects peripheral fission, we transfected N2a cells with the dephosphorylation-mimic mutant DRP1 S616A (Supplementary Figures S6B, C) or the phosphomimetic mutant DRP1 S637D (Supplementary Figures S6D, E). Live-cell time-lapse imaging showed that DRP1 S637D (Figures 6I, J) and S616A (Figures 6K, L) both inhibited excessive mitochondrial peripheral fission induced by PrP^106−126^ in FIS1-overexpressing N2a cells. DRP1 is a GTPase whose hydrolysis drives its self-assembly into ring-like structures, facilitating mitochondrial fission. DRP1 GTPase activity assays showed that DRP1 GTPase activity was enhanced following PrP^106−126^ treatment (Figure 6H). To explore the effect of DRP1 GTP hydrolysis on peripheral fission, we generated an enzymatically inactive DRP1 K38A mutant (Supplementary Figures S6F, G) in N2a cells. The results showed that DRP1 K38A attenuated the increased mitochondrial peripheral fission rate induced by PrP^106−126^ in FIS1-overexpressing N2a cells (Figures 6I, J). These results suggest that DRP1 activation through phosphorylation changes and GTPase activity is required for peripheral mitochondrial fission in prion disease.

### FIS1 inhibition alleviates mitochondrial morphological damage and dysfunction in the prion disease model

**Figure 7:**
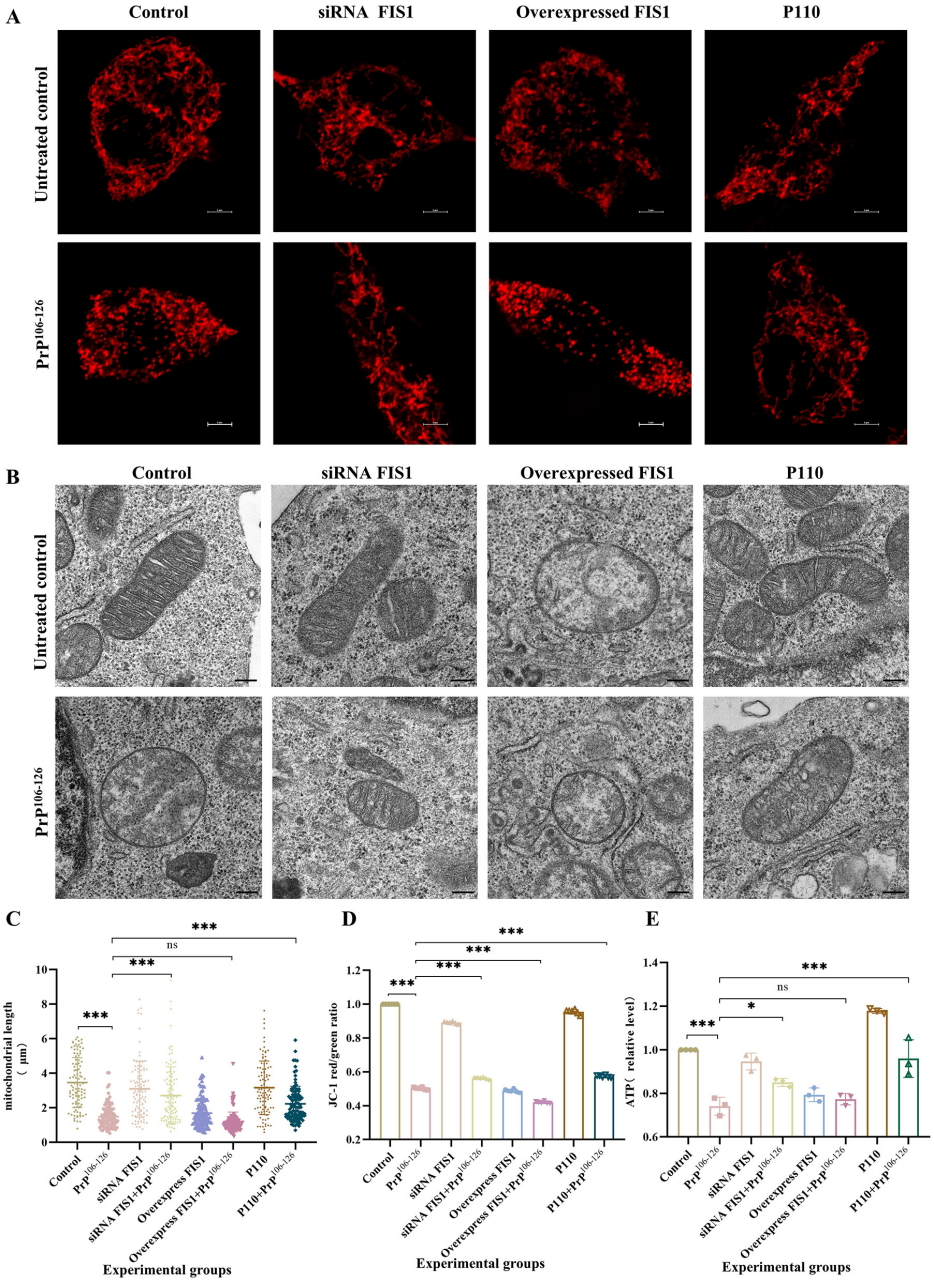
FIS1 inhibition alleviates mitochondrial morphological damage and dysfunction in the prion disease model. **(A)** Mitochondrial morphology was measured using confocal microscopy in knocked down (siRNA) FIS1, overexpressed FIS1, or P110-treated cells with or without PrP^106−126^ treatment. Scale bar: 10 μm. **(B)** Mitochondrial ultrastructure of cells observed using transmission electron microscopy (TEM). Scale bar: 200 nm. **(C)** Comparison of the mitochondrial length in knocked down (siRNA) FIS1 and overexpressed FIS1 cells with or without PrP^106−126^ and P110 treatments. **(D)** Detection of mitochondrial membrane potential (MMP) with JC-1 staining solution, as measured by changes in red/green intensity ratios, in knocked down (siRNA) FIS1 and overexpressed FIS1 cells with or without PrP^106−126^ and P110 treatments. **(E)** Detection of ATP levels in knocked down (siRNA) FIS1 and overexpressed FIS1 cells with or without PrP^106−126^ and P110 treatments. The data are presented as the mean ± SD. ns not significant; *P < 0.05; ***P < 0.001. *n* = at least three biologically independent treatments of cells for each.

Both *in vivo* and *in vitro* prion disease models have shown reduced ATP levels, mitochondrial membrane potential (MMP), cristae loss, and mitochondrial fragmentation (Li et al., 2018; Wu et al., 2019). Therefore, we aimed to determine whether inhibiting peripheral fission would alleviate mitochondrial morphological changes and dysfunction in N2a cells. Confocal imaging showed longer mitochondria in N2a cells after FIS1 knockdown or P110 treatment in the prion disease model (Figures 7A, C). We further examined the mitochondrial ultrastructure by transmission electron microscopy (TEM). Mitochondria in neurons treated with PrP^106−126^ or overexpressing FIS1 exhibited increased swelling and cristae loss. In contrast, mitochondria displayed remodeled cristae and alleviated swelling after FIS1 knockdown or P110 treatment in PrP^106−126^-treated N2a cells (Figure 7B). These findings suggest that FIS1 inhibition or P110 treatment alleviates mitochondrial fragmentation, swelling, and cristae loss induced by PrP^106−126^.

We next sought to explore whether alleviating mitochondrial morphological damage could mitigate mitochondrial dysfunction in the prion disease model. We measured mitochondrial membrane potential (MMP) and intracellular ATP levels and found that both were reduced after PrP^106−126^ treatment. FIS1 knockdown or P110 treatment prevented the reduction of MMP and ATP, suggesting that FIS1 inhibition or P110 treatment alleviatesPrP^106−126^-induced mitochondrial dysfunction (Figures 7D, E; Supplementary Figure S7). These combined results demonstrate that excessive mitochondrial peripheral fission leads to mitochondrial morphological damage and subsequent mitochondrial dysfunction. In conclusion, FIS1-mediated excessive mitochondrial peripheral fission exacerbates mitochondrial morphological changes and dysfunction in *in vitro* prion disease models, which can be alleviated by FIS1 knockdown or P110 treatment.

### FIS1 inhibition attenuates neuronal apoptosis in the prion disease model

**Figure 8:**
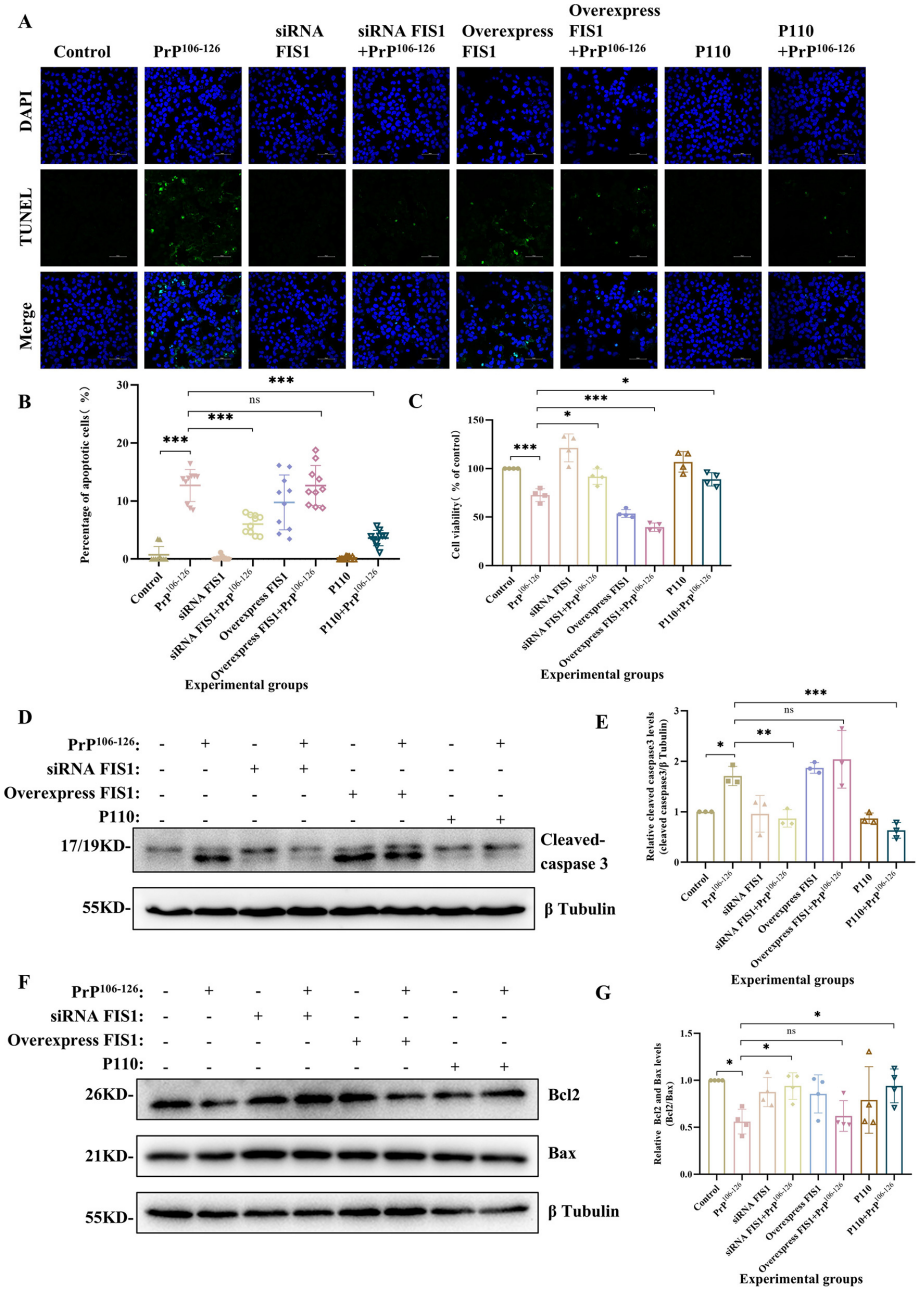
FIS1 inhibition attenuates neuronal apoptosis in the prion disease model. **(A)** Fluorescence images of cell apoptosis detected using TUNEL assays (green stain) in knocked down (siRNA) FIS1 and overexpressed FIS1 N2a cells with or without PrP^106−126^ and P110 treatments. Scale bar: 50 μm. **(B)** Comparison of the proportions of apoptotic cells to all cells in each group. **(C)** Detection of cell viability using the CCK-8 kit in knocked down (siRNA) FIS1 and overexpressed FIS1 N2a cells with or without PrP^106−126^ and P110 treatments. **(D)** Western blotting detection of the cleaved caspase-3 in knocked down (siRNA) FIS1 and overexpressed FIS1 N2a cells with or without PrP^106−126^ and P110 treatments. β Tubulin was used as the loading control. **(E)** Comparison of cleaved caspase-3 protein levels, relative to control levels. **(F)** Western blotting detection of Bcl2 and BAX levels in knocked down (siRNA) FIS1 and overexpressed FIS1 N2a cells with or without PrP^106−126^ and P110 treatments. β Tubulin was used as the loading control. **(G)** Comparison of Bcl2/Bax ratio. The data are presented as the mean ± SD. ns, not significant; *p < 0.05; **p < 0.01; ***p < 0.001. *n* = at least three biologically independent treatments of cells for each.

Previous studies have showed that PrP^106−126^-treated N2a cells undergo apoptosis. Inhibiting DRP1-mediated mitochondrial fission can reduce PrP^106−126^-induced apoptosis (Li et al., 2018). We aimed to investigate whether FIS1 modulates apoptosis. We transfected N2a cells with siRNA targeting FIS1 or a FIS1 overexpression plasmid, or treated the cells with P110. TUNEL staining showed that apoptosis increased after PrP^106−126^ treatment, and inhibition of peripheral fission via FIS1 knockdown or P110 treatment alleviated this PrP^106−126^-induced apoptosis in N2a cells (Figures 8A, B). Cell viability assays showed that PrP^106−126^ treatment reduced cell viability, whereas FIS1 knockdown or P110 treatment alleviated this reduction (Figure 8C). These findings demonstrate that inhibiting FIS1-mediated mitochondrial peripheral fission reduces neuronal apoptosis in a prion disease model.

Damaged mitochondria activate caspase-3, triggering mitochondrial pathway-dependent apoptosis, while Bcl-2 family proteins are key regulators of the release of mitochondria-associated apoptotic factors. We then measured intracellular levels of activated caspase-3 and the Bcl2/Bax ratio. Western blot analysis showed that PrP^106−126^ treatment increased activated caspase-3 levels and decreased the Bcl2/Bax ratio, whereas FIS1 knockdown or P110 treatment alleviated PrP^106−126^-induced caspase-3 activation and prevented the decrease in the Bcl2/Bax ratio (Figures 8D–G). These results demonstrate that excessive mitochondrial peripheral fission activates apoptosis via the mitochondrial pathway, and FIS1 inhibition alleviates PrP^106−126^-induced neuronal apoptosis.

## Discussion

Neurons rely on functional mitochondria to sustain normal physiological functions. Mitochondrial function is associated with various physiological processes, including mitochondrial dynamics, metabolism, mitophagy, and biogenesis (Harrington et al., 2023). Mitochondrial dynamics regulate ATP production to meet the demands of stem cell development, self-renewal, differentiation, and cellular adaptability (Zacharioudakis and Gavathiotis, 2023). In particular, mitochondrial fission responds to metabolic activation under different conditions. It ensures an adequate number of mitochondria for cell proliferation through MFF-mediated midzone fission and removes damaged mitochondria to maintain quality via FIS1-mediated peripheral fission (Tábara et al., 2025). In the present study, mitochondrial fragmentation, functional impairment, and apoptosis were detected in N2a cells treated with PrP^106−126^, which correlated with excessive mitochondrial peripheral fission. Our studies also indicate that excessive mitochondrial peripheral fission in prion *in vitro* models is mediated by FIS1, consistent with the literature (Kleele et al., 2021). We found that targeted modulation of FIS1 partially restores mitochondrial function and mitigates cell death, suggesting that peripheral fission plays a pivotal role in the pathogenesis of prion diseases and may serve as a potential therapeutic target. Furthermore, transcriptomic analysis revealed not only changes in FIS1 but also an increase in the mRNA levels of the uncoupling protein 2 (UCP2). Existing literature suggests that UCP2 regulates systemic glucose homeostasis in ventromedial hypothalamic nucleus neurons by modulating mitochondrial fission (Toda et al., 2016). This implies an adaptive mechanism through which UCP2 responds to metabolic changes and suggests that prp^106−126^ treatment may induce cellular metabolic alterations.

Recent studies on neurodegenerative diseases suggest that an imbalance in mitochondrial-lysosomal contacts plays a critical role in regulating neuronal homeostasis and contributing to disease progression (Cisneros et al., 2022). Mutations in the E3 ubiquitin ligase Parkin, which is linked to early-onset Parkinson's disease, reduce RAB7 activity, decrease mitochondrial-lysosomal contacts, and impair amino acid transfer from lysosomes to mitochondria (Peng et al., 2023). However, PD patients carrying the GBA1 mutation show reduced activity of the lysosomal enzyme β-glucocerebrosidase (GCase) and lower levels of TBC1D15, resulting in prolonged mitochondrial-lysosomal contacts (Kim et al., 2021). This demonstrates that the balance of organelle contacts is crucial for maintaining neuronal homeostasis and represents a key site of functional impairment in the early stages of neurodegeneration. Therefore, elucidating changes in mitochondrial-lysosomal contacts within prion disease models is crucial. We demonstrate that in prion diseases, the duration of mitochondrial-lysosomal contacts mediated by RAB7 GTP is reduced. As mitochondrial peripheral fission occurs after M-L contact, we investigated the relationship between peripheral fission and mitochondrial-lysosome contacts in prion disease models. Our results indicate that FIS1 enhances the recruitment of TBC1D15 to mitochondria. Modulating TBC1D15 to regulate RAB7 GTP hydrolysis affects the rate of mitochondrial peripheral fission. This finding further confirms that FIS1 mediates excessive peripheral fission by recruiting TBC1D15 to mitochondria, regulating RAB7 GTP hydrolysis, and accelerating the untethering of mitochondrial-lysosome contacts in prion disease models. During myocardial ischemia-reperfusion injury, TBC1D15 regulates mitochondrial asymmetric fission by modulating mitochondrial-lysosomal contact, consistent with our findings (Sun et al., 2022). We also expressed different RAB7 mutants and measured the fission rate. However, we found that expressing RAB7 T22N reduced the fission rate. Literature indicates that lysosomes transfer phosphatidylinositol-4-phosphate (PI(4)P) to mitochondria via mitochondrial-lysosome contacts to facilitate subsequent fission. Moreover, expression of both RAB7 Q67L and RAB7 T22N reduced the rate of mitochondrial fission compared to HeLa cells expressing RAB7 WT, consistent with our findings (Boutry and Kim, 2021). We surmise that RAB7 T22N expression decreases the duration of mitochondrial-lysosome contacts, affecting PI(4)P transfer and reducing the peripheral fission rate. These findings emphasize the importance of maintaining proper mitochondrial-lysosomal contacts. Collectively, these findings highlight the importance of properly regulated mitochondrial–lysosomal contact dynamics in controlling mitochondrial peripheral fission. Given that peripheral fission represents an early event in mitochondrial quality control, excessive FIS1-dependent peripheral fission under pathological conditions may ultimately influence the fate of damaged mitochondrial fragments.

As an early event in mitochondrial quality control, mitochondrial peripheral fission is functionally coupled to downstream mitophagy, which is responsible for the selective removal of damaged mitochondria and maintenance of neuronal homeostasis. Under physiological conditions, mitochondrial peripheral fission has been proposed to serve as an upstream segregation step that selectively isolates damaged mitochondrial portions from the intact mitochondrial network. These fragmented mitochondrial segments are enriched in proteins such as mitochondrial fission process 1 (MTFP1), which suppresses inner mitochondrial membrane fusion and prevent their reintegration into the healthy mitochondrial pool. Furthermore, the peripheral fission of mitochondria is closely related to mitochondrial autophagy, and it can promote the stability of PINK1, the recruitment of Parkin, and the assembly of autophagosomes in the early stage of autophagy initiation (Tábara et al., 2024; Mishra et al., 2025). Consistent with this coordinated process, the canonical PINK1–Parkin pathway plays a central role in recognizing depolarized mitochondria and targeting them for autophagic degradation, thereby ensuring efficient mitochondrial quality control (Palikaras et al., 2018).

However, accumulating evidence suggests that this functional coupling between mitochondrial fission and mitophagy is disrupted in prion disease. Previous studies have demonstrated impaired PINK1–Parkin-mediated mitophagy, characterized by reduced Parkin translocation to mitochondria, decreased LC3-associated mitochondrial clearance, and the accumulation of dysfunctional mitochondria, indicating a failure of mitochondrial quality control (Li et al., 2022). Therefore, although mitochondrial fission is enhanced in prion-treated cells, the subsequent clearance of damaged mitochondrial fragments appears to be insufficient, resulting in an uncoupling between mitochondrial fragmentation and mitophagic removal.

In this context, our findings suggest that excessive FIS1-mediated peripheral fission may further aggravate this imbalance by accelerating mitochondrial fragmentation without ensuring efficient downstream mitophagic clearance. Consequently, damaged mitochondrial fragments may accumulate rather than be eliminated, thereby exacerbating mitochondrial dysfunction and neuronal stress. Further studies are required to determine whether FIS1-dependent mitochondrial fission directly influences PINK1 stabilization and Parkin recruitment during prion-induced neurotoxicity.

FIS1 is an OMM protein that functions as a receptor for DRP1 on mitochondria (Atkins et al., 2016). In our *in vitro* prion disease model, FIS1 was found to recruit and interact with DRP1. We found that P110 treatment alleviated excessive peripheral fission and improved mitochondrial morphology in response to PrP^106−126^, demonstrating that FIS1 regulates peripheral fission by recruiting DRP1 to mitochondria in the prion disease model. Furthermore, FIS1 inhibition or P110 treatment restored mitochondrial function and reduced neuronal apoptosis induced PrP^106−126^. P110 is a selective inhibitor of mitochondrial fission. It specifically binds to the domain on DRP1 that interacts with FIS1, blocking the DRP1/FIS1 interaction without affecting interactions between DRP1 and other mitochondrial adaptor proteins (Qi et al., 2012). In various neurodegenerative disease models, including hereditary spastic paraplegia, traumatic brain injury, and Huntington's disease, P110 has been shown to mitigate DRP1-FIS1-mediated excessive mitochondrial fission and dysfunction. This finding is consistent with our results (Joshi et al., 2018, 2019). These results highlight the importance of FIS1 as a potential regulatory target in prion diseases and suggest the therapeutic potential of P110 for neurodegenerative disorders. However, the post-translational modifications (PTMs) of FIS1 remain unexplored. Under cellular stress, Fis1 Thr34 is phosphorylated by DNA-dependent protein kinase 1 (DNA-PKcs; Wang et al., 2022), whereas in cancer cells, phosphorylation occurs at Tyr38 mediated by Met kinase (Yu et al., 2021). This phosphorylation induces a conformational change in FIS1, triggering Cys41-mediated covalent homodimerization, which facilitates the recruitment of DRP1 to mitochondria (Pokhrel et al., 2025). Future studies should investigate FIS1 phosphorylation alterations in prion disease models to better understand prion pathogenic mechanisms.

DRP1 is a GTPase that is inactive in the cytoplasm; however, when activated, DRP1 is transferred to the mitochondria for subsequent fission. Previous studies in our laboratory showed a significant increase in DRP1 protein levels on mitochondria in both *in vitro* and *in vivo* prion disease models (Li et al., 2018). However, the role of DRP1 activity in disease models remains unexplored. DRP1 activity is regulated by multiple PTMs. Among these PTMs, phosphorylation at S616 and dephosphorylation at S637 have been extensively studied, with associations reported for DRP1 mitochondrial localization (Cereghetti et al., 2008; Kashatus et al., 2011). Our study showed increased DRP1 S616 phosphorylation and S637 dephosphorylation in the prion disease model. DRP1 S616 is phosphorylated by various kinases, including cyclin-dependent kinase (CDK)-1, extracellular signal-regulated kinases 1/2, protein kinase C isoform delta, and CDK5 (Zaja et al., 2014). Conversely, the DRP1 S637 site is dephosphorylated by calcineurin (Cereghetti et al., 2008) and PGAM5 (Yu et al., 2020). Phosphorylation at S616 or dephosphorylation at S637 enhances DRP1 activity, promoting mitochondrial fission. These observations indicate that DRP1 phosphorylation is primarily regulated by upstream kinase/phosphatase signaling rather than by mitochondrial adaptor proteins such as FIS1. Literature reports indicate that cellular calcium ion levels are elevated in prion models (Moon and Park, 2020). As calcium ions can activate calcineurin, we hypothesized that DRP1 S637 is dephosphorylated by calcineurin. Our experimental results show that in prion models, the DRP1 protein undergoes dephosphorylation at the S637 site by calcineurin, subsequently leading to its transport to the mitochondria. In an LPS-induced microglial activation model, peroxiredoxin 5 regulates mitochondrial fission by modulating Ca^2^?/calcineurin-dependent DRP1 S637 dephosphorylation through the elimination of cytoplasmic reactive oxygen species, consistent with our findings (Park et al., 2016). Inhibition of DRP1 activation by expressing DRP1 S637D or S616A reduced excessive mitochondrial peripheral fission caused by PrP^106−126^ in FIS1-overexpressing N2a cells. These results illustrate that DRP1 localization to mitochondria for peripheral fission requires S616 phosphorylation and S637 dephosphorylation. Together, our findings support a model in which FIS1 facilitates DRP1 mitochondrial recruitment, while DRP1 activation is regulated by upstream kinase/phosphatase signaling. Whether FIS1 indirectly influences DRP1 phosphorylation through these pathways remains an interesting question for future investigation.

Once recruited to the mitochondria, DRP1 mediates mitochondrial fission through its GTPase activity, inducing DRP1 filaments on the OMM to curl into loops, constricting and severing the mitochondrial membrane (Rochon et al., 2024). We observed increased DRP1 GTPase activity in prion models, and overexpression of the DRP1 K38A mutant reduced excessive mitochondrial peripheral fission caused by PrP^106−126^ in FIS1-overexpressing N2a cells, demonstrating the necessity of DRP1 GTPase activity for peripheral fission. Additionally, our experimental results show that inactive DRP1 inhibits peripheral fission even in cells expressing FIS1, providing further evidence that FIS1 requires active DRP1 to perform fission.

Although the PrP^106−126^-treated N2a cell model has been widely used to investigate prion-associated neurotoxicity and provides unique advantages for mechanistic studies of mitochondrial peripheral fission, particularly when combined with live-cell imaging, it primarily models the early neurotoxic effects of prion peptides rather than authentic prion propagation. To assess the broader applicability of our findings, we additionally examined key molecular alterations in human SH-SY5Y neuroblastoma cells and observed similar increases in mitochondrial FIS1 expression, unchanged MFF levels, increased DRP1 Ser616 phosphorylation, and decreased DRP1 Ser637 phosphorylation following PrP^106−126^ treatment, suggesting that the FIS1-associated molecular response is reproducible across distinct neuronal cell lines. Nevertheless, future studies using bona fide prion-infected cell lines and animal models will be necessary to determine whether FIS1-mediated mitochondrial peripheral fission represents a general pathogenic mechanism during prion disease progression and to further evaluate its potential as a therapeutic target.

In summary, our study demonstrates that FIS1 mediates excessive mitochondrial peripheral fission by regulating mitochondria–lysosome contact dynamics and recruiting DRP1 in a PrP^106−126^-induced neuronal injury model, thereby exacerbating mitochondrial fragmentation, dysfunction, and apoptosis. Inhibition of FIS1-mediated peripheral fission, either by genetic knockdown or pharmacological disruption of the FIS1/DRP1 interaction, effectively alleviated mitochondrial damage and neuronal apoptosis. Collectively, our findings establish a molecular framework linking aberrant mitochondrial peripheral fission to early prion-associated neurotoxicity and provide a rationale for further investigating FIS1-mediated peripheral fission as a potential therapeutic target in authentic prion disease models and other neurodegenerative disorders. Furthermore, our study advances the understanding of how dynamic organelle interactions regulate mitochondrial networks and contribute to neuronal dysfunction.

## Materials and methods

### Cell culture

Mouse neuroblastoma N2a cells (ATCC, Manassas, VA, USA, CCL-131) were obtained from the Cell Resource Center of Peking Union Medical College and confirmed to be mycoplasma-free. Cells were maintained in Gibco DMEM (Thermo Fisher Scientific, Waltham, MA, USA, C11995500BT) supplemented with 10% (v/v) fetal bovine serum (FBS; Gibco, Thermo Fisher Scientific) under standard culture conditions at 37 °C in a humidified atmosphere containing 5% CO_2_. Human neuroblastoma SH-SY5Y cells were purchased from Wuhan Pricella Biotechnology Co., Ltd. (CL-0208). Cells were cultured in DMEM/F12 medium (HUANKE, HK2109.17) supplemented with 10% fetal bovine serum (NEWZERUM, FBS-E500) and 1% penicillin-streptomycin (Beyotime, C0222) at 37 °C in a humidified incubator containing 5% CO_2_. All cell lines were routinely tested and confirmed to be free of mycoplasma contamination.

The PrP^106−126^ peptide (KTNMKHMAGAAAAGAVVGGLG; >98% purity) and its scrambled control sequence (MEVGWYRSPFSRVVHLYRNGK) were synthesized by Amy Peptides Bio-Tech. Both peptides were dissolved in PBS at 1 mM and incubated with agitation at 4 °C for 24 h to allow aggregation. The working concentration used in experiments was 100 μM. All procedures were conducted under sterile conditions.

P110 (Selleck, Houston, TX, USA, S9887) was prepared in water as a 10 mg/mL stock solution and stored at −20 °C, with a working concentration of 1 μM used in experiments. Cyclosporin A (CsA; Selleck, S2286) was dissolved in ethanol to the same stock concentration (10 mg/mL) and stored at −20 °C, and was applied at a final concentration of 2 μM.

### Mitochondrial isolation

Mitochondrial and cytosolic proteins were isolated from N2a or SH-SY5Y cells using the Cell Mitochondrial Isolation Kit (C3601, Beyotime Biotechnology, Shanghai, China) according to the manufacturer's protocol. Collected cells were resuspended in mitochondrial isolation buffer supplemented with 1 mM PMSF (P0100, Solarbio Life Sciences, Beijing, China) for 15 min. Cells were homogenized 30 times and centrifuged at 1,000 × g for 10 min at 4 °C. The resulting supernatant was further centrifuged at 11,000 × g for 10 min at 4 °C. The mitochondrial pellet was finally resuspended in lysis buffer containing 1 mM PMSF for subsequent Western blot analysis.

### RNA sequencing

Total RNA was isolated from N2a cells using TRIzol^®^ Reagent in accordance with the manufacturer's protocol. RNA integrity was assessed using the Agilent 5300 Bioanalyzer, and RNA concentration was measured with an ND-2000 NanoDrop spectrophotometer (NanoDrop Technologies). Only samples meeting quality criteria (concentration ≥ 20 ng/μL, total RNA > 1 μg, RQN > 4.5) were used for sequencing library construction. 1 μg of RNA was used for Illumina mRNA library preparation, and sequencing was performed on the NovaSeq X Plus or DNBSEQ-T7 platform. 10 ng of RNA was used for SMART-Seq V4 library preparation, with sequencing on the same platforms. Raw data was trimmed using fastp, mapped to the reference genome using HISAT2, and assembled with StringTie. Differentially expressed genes (Fold change ≥ 1.5, FDR < 0.05) were identified using DESeq2 or DEGseq, followed by GO enrichment analysis. Alternative splicing eventswere identified using rMATS.

### Immunofluorescence staining

N2a cells were transfected with DsRed-Mito plasmid or siRNA and incubated for 48 h before exposure to PrP^106−126^. Cells were gently washed twice with PBS and fixed for 30 min using a fixation solution for immunostaining (P0098, Beyotime Biotechnology, Shanghai, China). Permeabilization was then performed with a Triton X-100 which contains fixation/permeabilization buffer (P0096, Beyotime Biotechnology) for 5 min at room temperature. Cells were subsequently blocked with blocking buffer (P0102, Beyotime Biotechnology) for 1 h at room temperature, followed by incubation with primary antibodies overnight at 4 °C and secondary antibodies for 1 h at room temperature. After washing, the slides were mounted with an anti-quenching agent containing DAPI (S2110, Solarbio Life Sciences, Beijing, China). Immunofluorescence images were captured with a Nikon A1 confocal microscope (Nikon, Tokyo, Japan). The Pearson correlation coefficient was calculated using ImageJ software.

### ATP level measurement

N2a cells were completely lysed, and ATP content was measured using an Enhanced ATP Assay Kit (S0027, Beyotime Biotechnology, Shanghai, China) in accordance with the manufacturer's protocol. Luminescence signals were subsequently detected with a GloMax 96 microplate luminometer (Promega, Madison, WI, USA).

### Mitochondrial membrane potential level detection

The MMP Assay Kit (C2006, Beyotime Biotechnology, Shanghai, China) was assessed using the MMP Assay Kit (C2006, Beyotime Biotechnology, Shanghai, China) following the manufacturer's protocol. N2a cells were incubated with JC-1 staining solution for 20 min at 37 °C in a 5% CO_2_ incubator, after which MMP was promptly measured. Fluorescence signals were detected with a FACSCalibur flow cytometer (BD Biosciences, San Jose, CA, USA).

### TUNEL assay

Apoptosis in N2a cells was assessed using the One-Step Apoptosis Assay Kit (C1086, Beyotime Biotechnology, Shanghai, China) following the manufacturer's protocol. Cells were imaged using a Nikon A1 confocal microscope (Nikon, Tokyo, Japan).

### Cell viability assay

N2a cell viability was evaluated using the Cell Counting Kit-8 (CCK-8; C0038, Beyotime Biotechnology, Shanghai, China) following the manufacturer's protocol. Absorbance was recorded at 450 nm with a BioTek microplate reader (BioTek, Manchester, NH, USA), and cell viability was calculated relative to the untreated control group..

### DRP1 GTPase activity assay

N2a cells were lysed and incubated with Drp1 antibody at 4 °C for 5 h. Next, 50 μL of protein A/G agarose beads were added to each sample, followed by overnight immunoprecipitation at 4 °C. After incubation of the protein–antibody complexes, the beads were collected by centrifugation and washed twice before being subjected to analysis with a DRP1 GTPase activity assay kit (P2435S, Beyotime Biotechnology, Shanghai, China). The absorbance of the samples was measured at a wavelength of 630 nm using a microplate reader (BioTek, Manchester, NH, USA).

### Transmission electron microscopy

N2a cells were harvested by centrifugation at 800 rpm for 4 min and fixed in 2.5% glutaraldehyde at 4 °C for 12 h. Subsequently, the samples were embedded, and mitochondrial morphology was examined and quantified using a Hitachi HT7700 transmission electron microscope (TEM, Tokyo, Japan).

### Live cell time-lapse imaging

N2a cells were transferred into a glass dish at least 12 h prior to imaging. To stain the mitochondria, the cells were incubated at 37 °C for 5 min with 500 nM Invitrogen Mito-Tracker Green FM (ThermoFisher Scientific, Waltham, MA, USA, M7514) diluted with DEME. For lysosomal staining, cells were incubated with 50 nM LysoTracker Red (C1046, Beyotime Biotechnology, Shanghai, China) diluted in DMEM at 37 °C for 20 min. After staining, the cells were washed gently with PBS three times, and then the Invitrogen Live Cell Imaging Solution (ThermoFisher Scientific, Waltham, MA, USA, A59688DJ) with Invitrogen ProLong Live Antifade Reagent (ThermoFisher Scientific, Waltham, MA, USA, P36975) was added to the glass dish. The LSM 880 Airyscan super-resolution confocal microscope (Zeiss, Oberkochen, Germany) was used for live cell delay imaging, and photographed 5 cells at a time for 5 min.

In living cells, the division of a single mitochondrion into two daughter mitochondria is considered a mitochondrial fission event. The lengths of parental and daughter mitochondria were measured using segmented lines. If the daughter's length exceeded 25% of the parent's, the fission event was classified as a midzone fission event. If the daughter's length was equal to or less than 25% of the parent's, the fission event was categorized as a peripheral fission event^25^. Based on previously published methods (Kleele et al., 2021; Liu et al., 2024), we defined the mitochondrial fission rate as the number of fission events per square micron of mitochondrial area. Mitochondrial area was measured using ImageJ thresholding. For live-cell imaging analyses, five cells were analyzed per biological replicate according to the predefined criteria described above. Based on previous research (Wong et al., 2018), the mitochondria–lysosome contacts were identified as those that showed mitochondria and lysosomes in close proximity (< 0.1 μm) for >10 s in time-lapse images in living cells.

### Data statistics and analysis

The data are presented as the mean ± standard deviation (SD) and were analyzed using Prism 8.0 software. A Student's *t*-test was used to compare differences between two groups. Differences between multiple groups were analyzed using a one-way ANOVA, followed by Dunnett's *post hoc* test. Statistical significance was considered at a level of *p* < 0.05. Statistical analyses were performed using Prism 8.0 software (GraphPad, La Jolla, CA, USA) or ImageJ (National Institutes of Health, USA).

## Data Availability

The datasets presented in this study can be found in online repositories. The names of the repository/repositories and accession number(s) can be found at: https://www.ncbi.nlm.nih.gov/, PRJNA1471176.
