# Supplementary material for: FIS1 dependent peripheral fission exacerbates mitochondrial fragmentation and dysfunction in prion diseases
Source: Front Neurosci. 2026 Aug 5;20:1893232. doi: 10.3389/fnins.2026.1893232 (PMC13486221; doi:10.3389/fnins.2026.1893232)
Supplement: Supplementary file 1 [file Data_Sheet_1.pdf]

Supplementary Figures

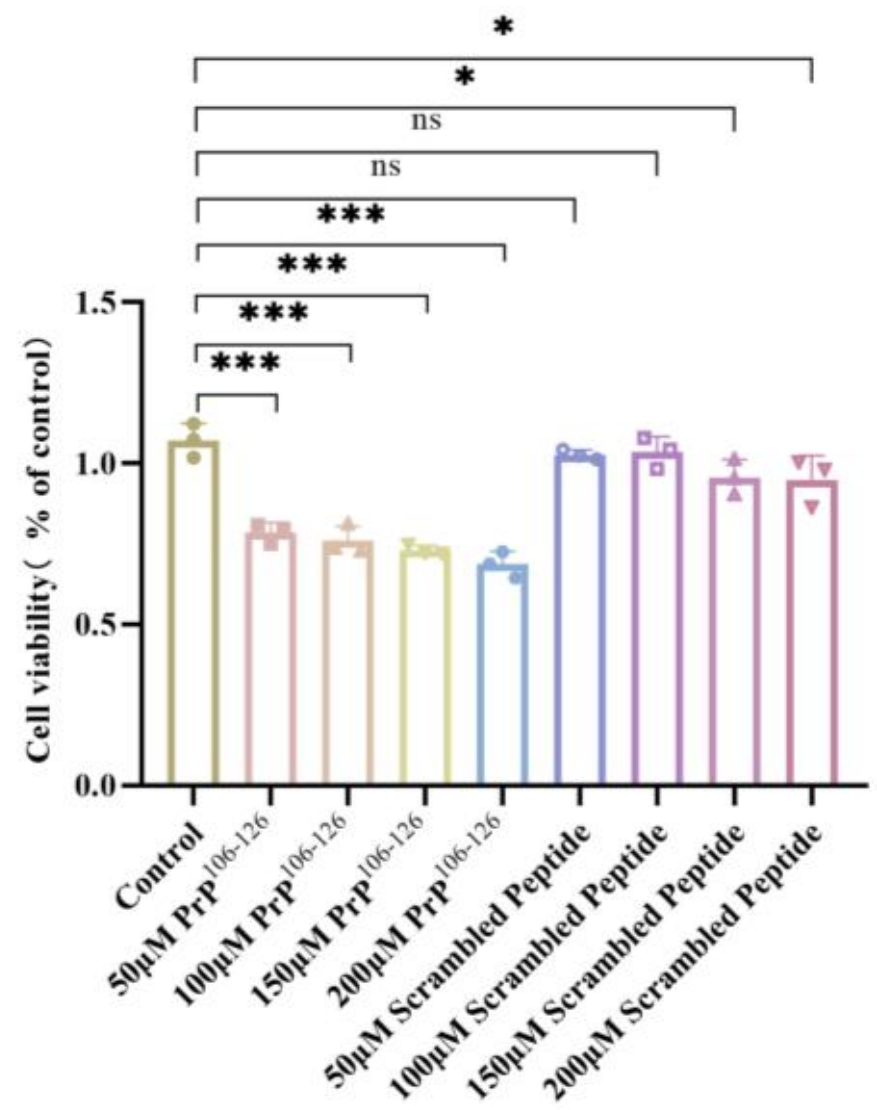

Supplementary Figure 1. Detection of cell viability using the CCK-8 kit in N2a cells with 50μM, 100μM，150μM, 200μM $PrP^{106–126}$ or Scrambled Peptide. Data are mean ± SD. ns, not significant; *$P < 0.05$; **$P < 0.01$; ***$P < 0.001$. n = at least 3 biologically independent treatments of cells for each.

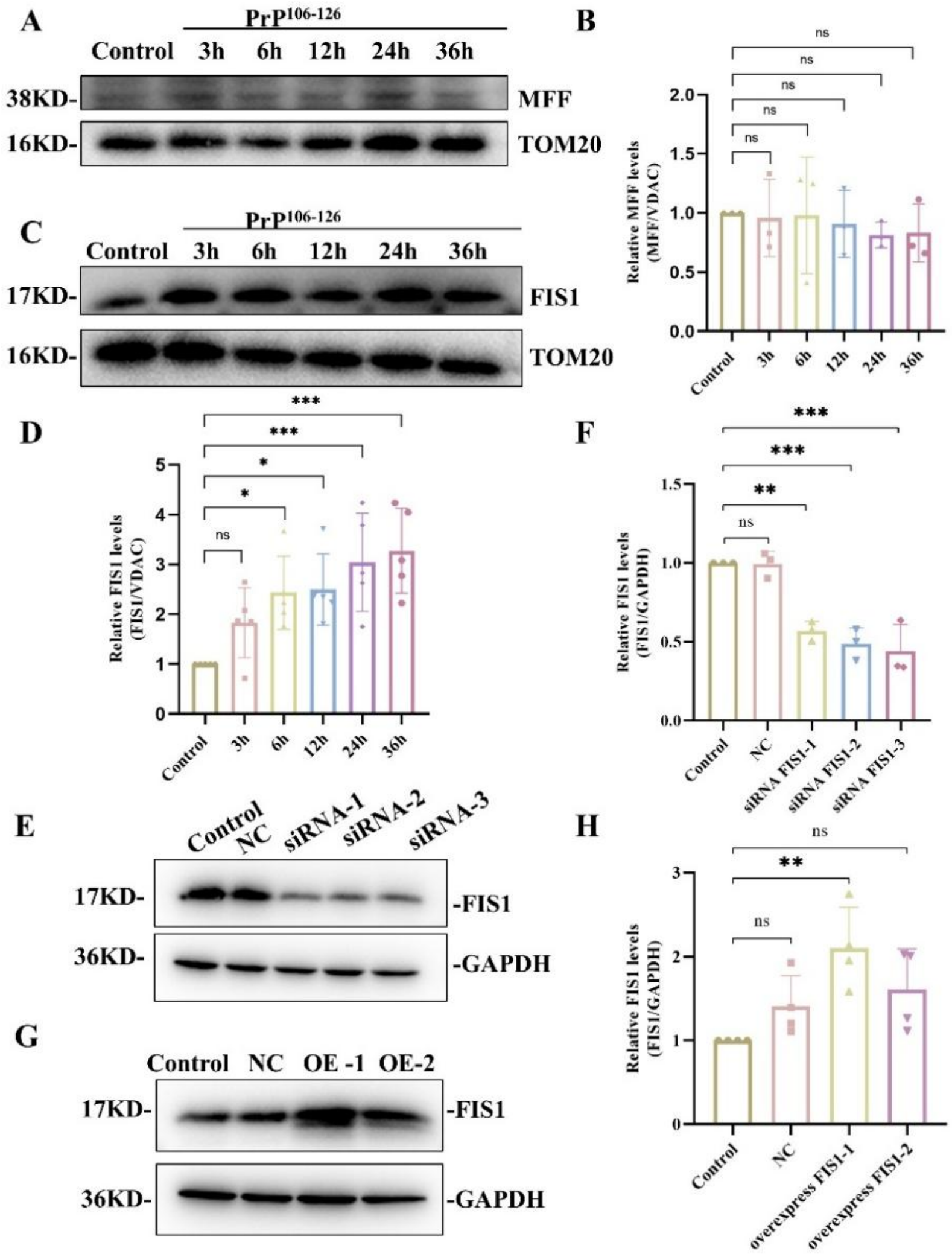

Supplementary Figure 2. A: Western blotting of mitochondrial MFF protein extracted from SH-SY5Y cells. TOM20 was used as the loading control. **B**: Comparison of MFF protein levels, relative to control. **C**: Western blotting of mitochondrial FIS1 protein extracted from SH-SY5Y cells. TOM20 was used as the loading control. **D**: Comparison of FIS1 protein levels, relative to control. **E**: Western blotting was used to detect knocked down (siRNA) FIS1 protein in N2a cells. GAPDH was used as the loading control. **F**: Comparison of FIS1 protein levels, relative to control. **G**: Western blotting was used to detect FIS1 overexpression in N2a cells. GAPDH was used as the loading control. **H**: Comparison of FIS1 protein levels, relative to control. Data are mean ± SD. ns, not significant; **P* < 0.05; ***P* < 0.01; ****P* < 0.001. n = at least 3 biologically independent treatments of cells for each.

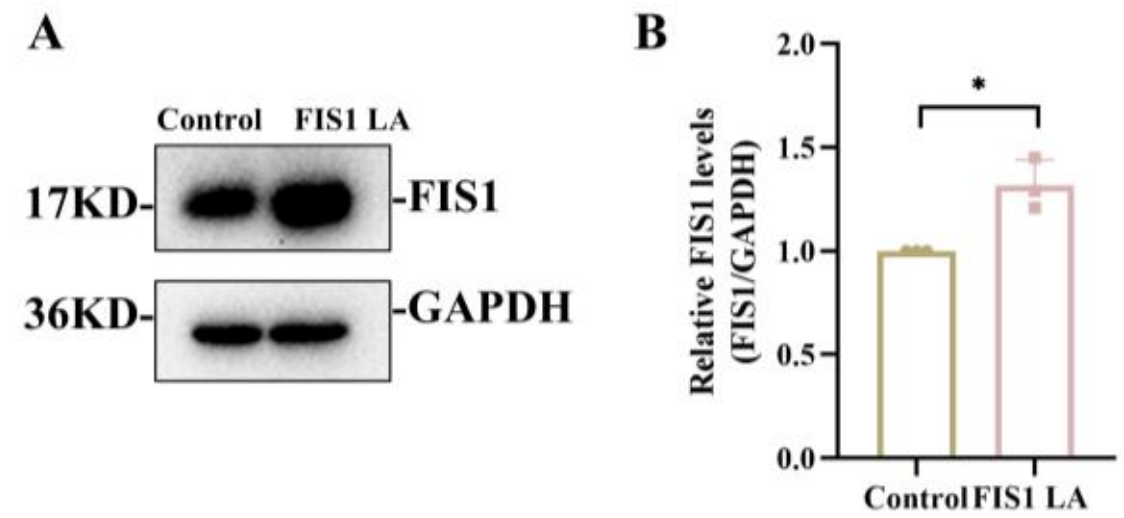

Supplementary Figure 3. A: Western blotting was used to detect FIS1overexpression in N2a cells. GAPDH was used as the loading control. **B**: Comparison of FIS1 protein levels, relative to control. Data are mean ± SD. ns, not significant; **P* < 0.05; ***P* < 0.01; ****P* < 0.001. n = at least 3 biologically independent treatments of cells for each.

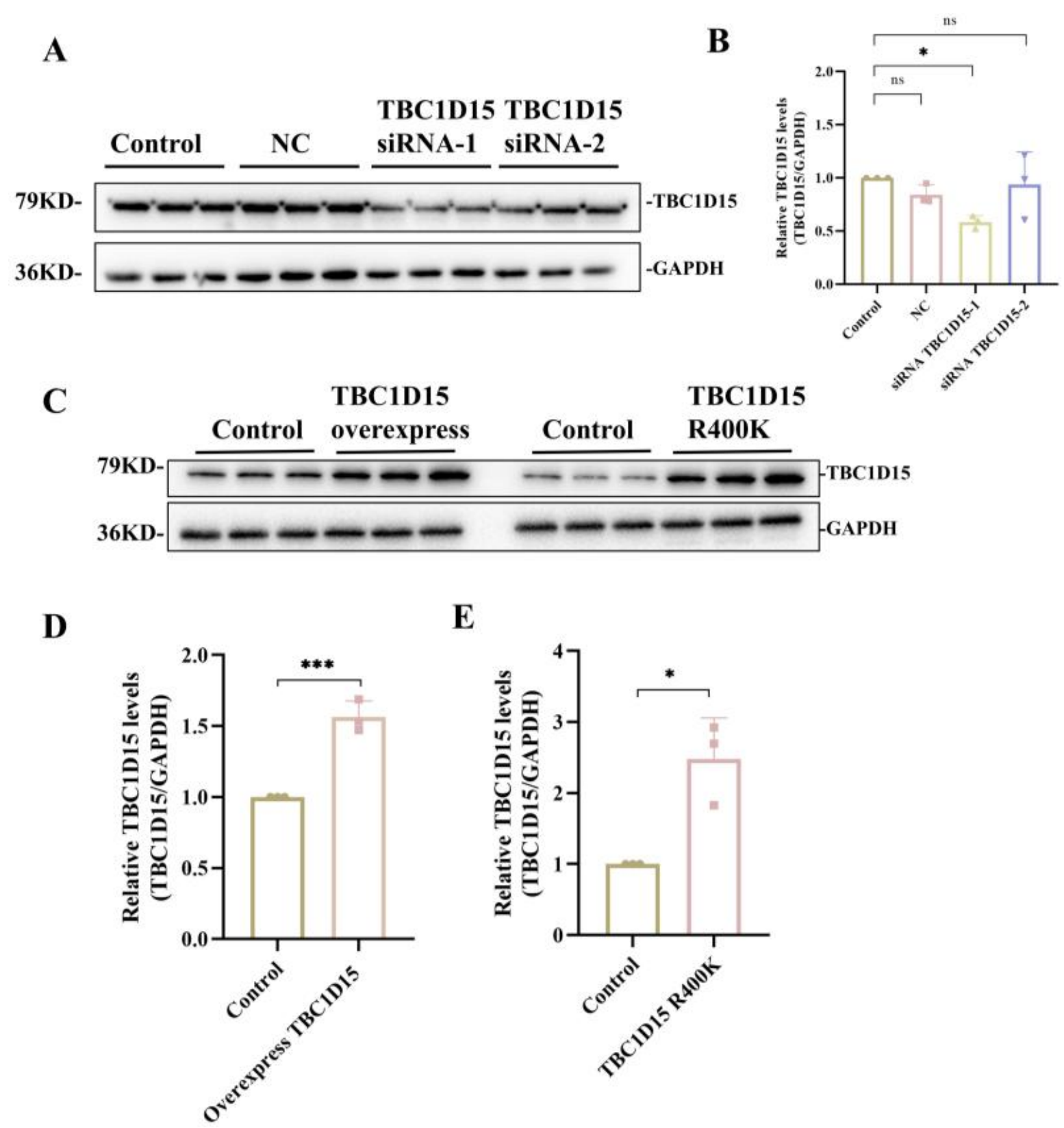

Supplementary Figure 4. A: Western blotting was used to detect knocked down (siRNA) TBC1D15 protein in N2a cells. GAPDH was used as the loading control. **B**: Comparison of TBC1D15 protein levels, relative to control. **C**: Western blotting was used to detect TBC1D15 overexpression in N2a cells. GAPDH was used as the loading control. **D, E**: Comparison of TBC1D15 protein levels, relative to control. Data are mean ± SD. ns, not significant; **P* < 0.05; ***P* < 0.01; ****P* < 0.001. n = at least 3 biologically independent treatments of cells for each.

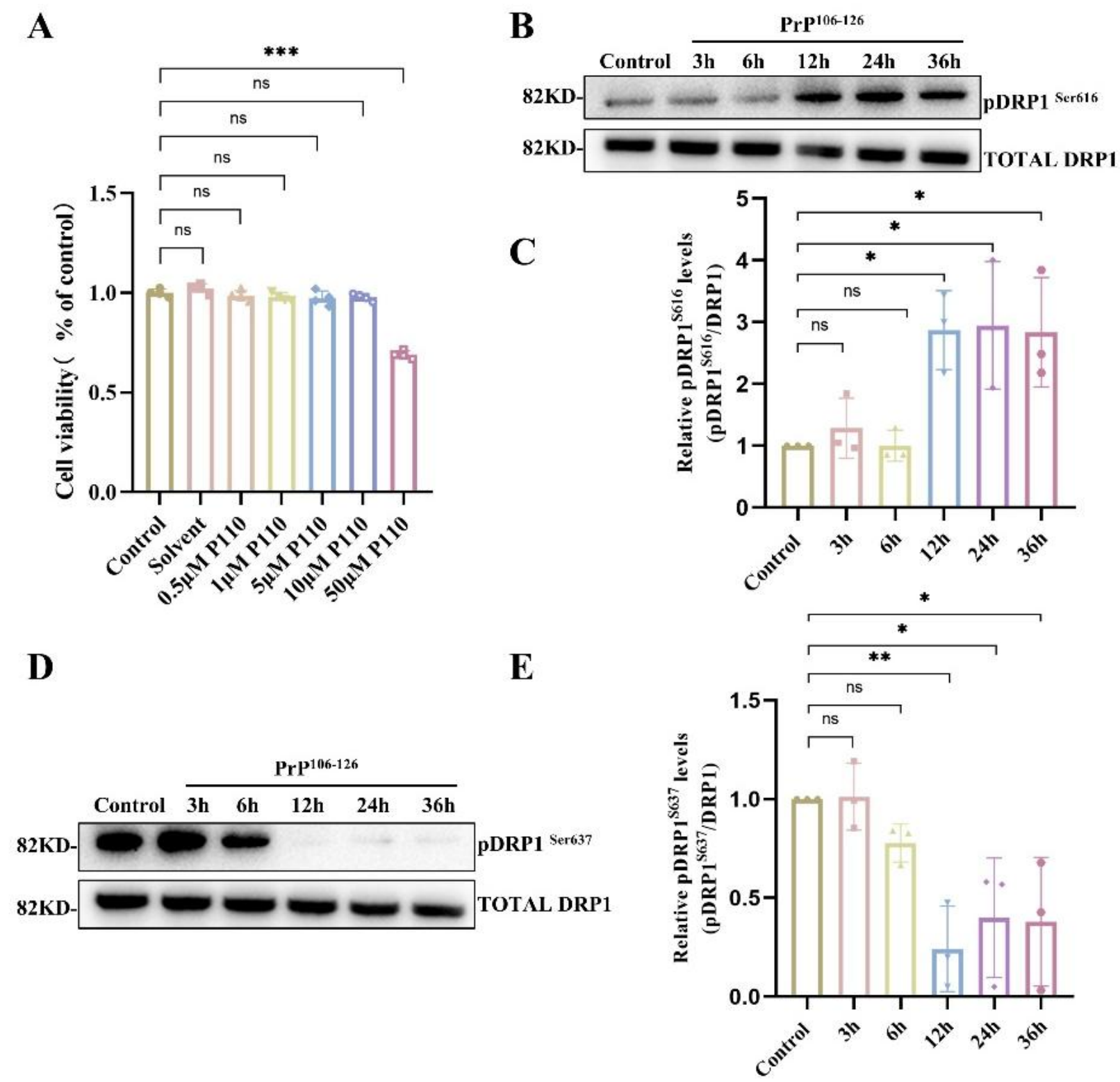

Supplementary Figure 5. A: Detection of cell viability using the CCK-8 kit in N2a cells with 0.5μM, 1μM， 5μM, 10μM, 50μM P110 treatment. **B**: Western blotting detection of phosphorylated DRP1 at sites S616 ($pDRP1^{S616}$) in SH-SY5Y cells treated with or without $PrP^{106–126}$. The total DRP1 protein was used as the loading control. **C**: Comparison of $pDRP1^{S616}$ protein levels, relative to control. **D**: Western blotting detection of phosphorylated DRP1 at sites S637 ($pDRP1^{S637}$) in SH-SY5Y cells treated with or without $PrP^{106–126}$. The total DRP1 protein was used as the loading control. **E**: Comparison of $pDRP1^{S637}$ protein levels, relative to control. Data are mean ± SD. ns, not significant; *P < 0.05; **P < 0.01; ***P < 0.001. n = at least 3 biologically independent treatments of cells for each.

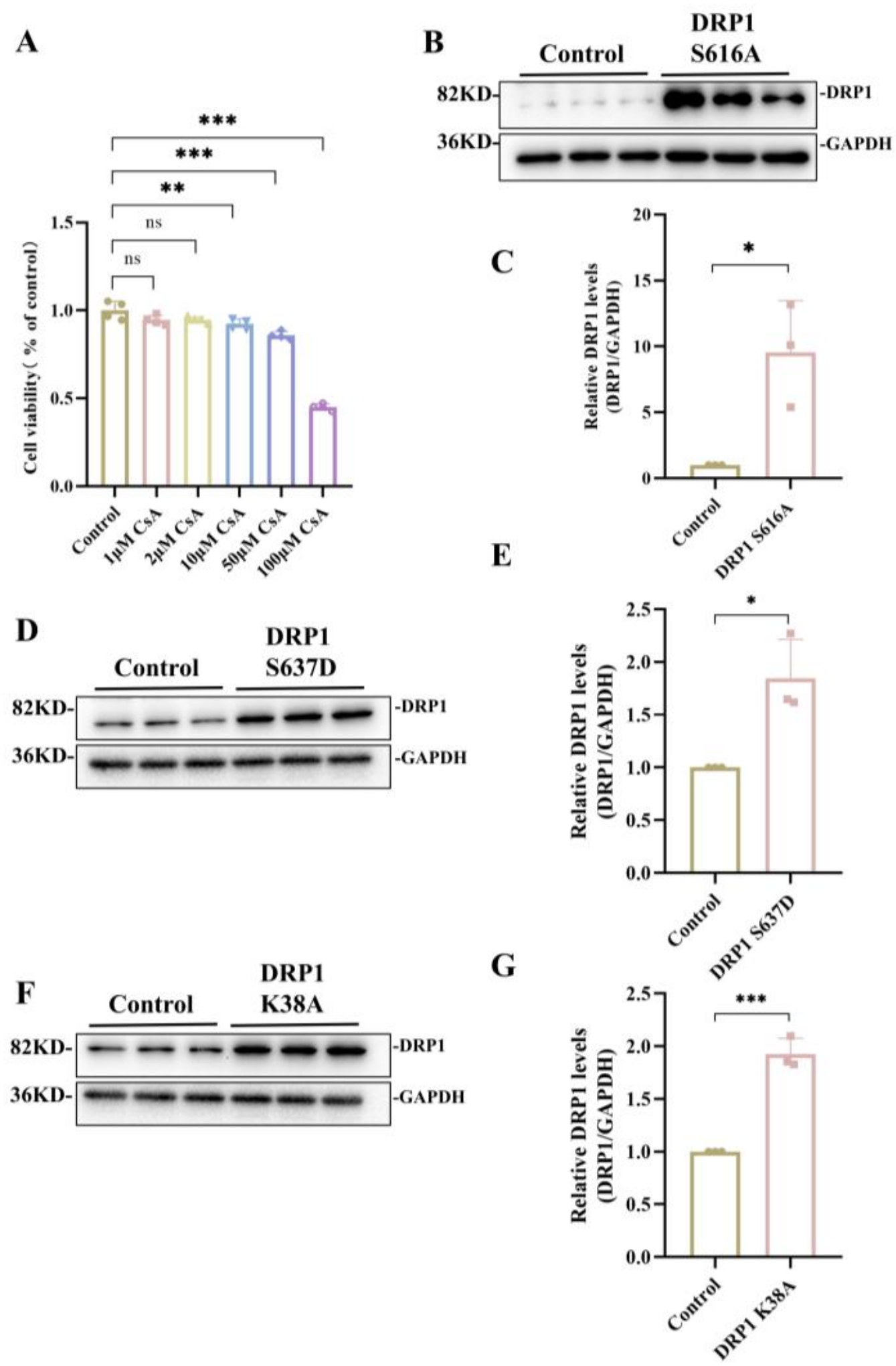

Supplementary Figure 6. A: Detection of cell viability using the CCK-8 kit in N2a cells with 1μM, 2μM, 10μM, 50Mm, 100μM CSA. **B, D, F**: Western blotting was used to detect DRP1 overexpression in N2a cells. GAPDH was used as the loading control. **C, E, G**: Comparison of DRP1 protein levels, relative to control. Data are mean ± SD. ns, not significant; *P < 0.05; **P < 0.01;***P < 0.001. n = at least 3 biologically independent treatments of cells for each.

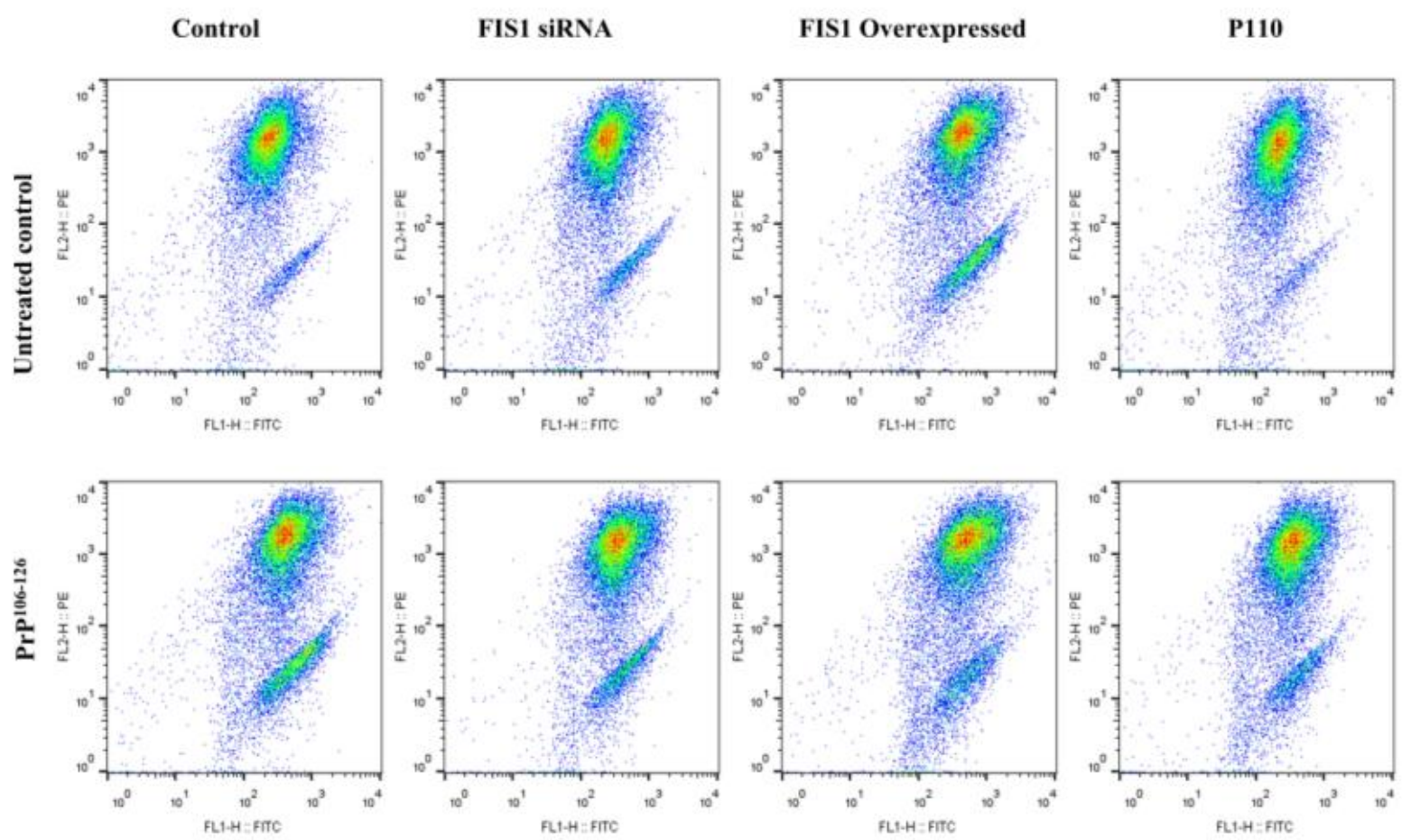

Supplementary Figure 7. FACS results of mitochondrial membrane potential (MMP) assays in knocked down (siRNA) FIS1 and overexpressed FIS1 cells with or without $PrP^{106–126}$ and P110 treatments. Data are mean ± SD. ns not significant; **P* < 0.05; ***P* < 0.01; ****P* < 0.001. n = at least 3 biologically independent treatments of cells for each.
